# Advances in Formulation and Technological Strategies for Montelukast Pulmonary Delivery

**DOI:** 10.1021/acsomega.6c03584

**Published:** 2026-07-24

**Authors:** Carla Giordani Testa, Millena de Sousa Afonso, João Vitor Vicente-da-Silva, Érika Yoko Suzuki, Renata Ribeiro de Castro, Luiz Cláudio Rodrigues Pereira da Silva, Lucio Mendes Cabral, Alice Simon, Flávia Almada Do Carmo

**Affiliations:** † Department of Drugs and Pharmaceutics, Faculty of Pharmacy, 314177 Universidade Federal Do Rio de Janeiro, 21941-902 Rio de Janeiro, Brazil; ‡ Brazilian Navy Pharmaceutical Laboratory, Marinha Do Brasil, 20911-291 Rio de Janeiro, Brazil; § Department of Pharmaceutical Sciences, Universidade Federal Rural Do Rio de Janeiro, 23897-090 Seropédica, Brazil; ∥ Instituto de Tecnologia Em Fármacos, Fundação Oswaldo Cruz, 21041-250 Rio de Janeiro, Brazil

## Abstract

Montelukast is a leukotriene receptor antagonist used in the management of asthma by modulating airway inflammation. Conventional oral formulations exhibit considerable variation in bioavailability due to extensive first-pass metabolism, short half-life, and wide distribution into peripheral compartments. These limitations, along with systemic adverse effects, challenge consistent therapeutic outcomes. Pulmonary drug delivery has been proposed as a promising alternative by targeting the drug directly to the lungs, potentially bypassing first-pass metabolism and reducing systemic exposure. However, despite extensive research on inhalable montelukast formulations, no product has yet been commercialized. This review discusses the main challenges involved in formulating montelukast for pulmonary administration and highlights recent advances in particle engineering, nanocarriers, and formulation strategies to overcome these limitations. Considering the potential advantages of pulmonary delivery for montelukast, a literature search was conducted to identify inhalable formulations of the drug. Twenty articles were identified, including sixteen on DPIs, one on nebulized montelukast, one on pMDIs and two articles reporting results related to clinical studies of inhaled montelukast. Overall, three clinical studies specifically evaluated inhaled montelukast. No patents evaluating inhaled montelukast were found, although three patents mention inhalation as a possible route without disclosing actual inhaled formulations or device strategies. The available studies were analyzed according to formulation strategies, particle engineering approaches, inhalation devices, and reported outcomes. For DPI formulations, particle aerodynamic diameter plays a critical role in lung deposition, and a mass median aerodynamic diameter (MMAD) in the range of 1–5 μm is generally considered optimal for lung deposition. After deposition, formulation performance depends on an initial burst release followed by sustained drug release, low epithelial permeability, and prolonged pulmonary retention, which are expected to minimize systemic exposure. Overall, the limited number of research highlights a translational gap and important opportunities for the development of inhalable montelukast therapies.

## Graphical abstract

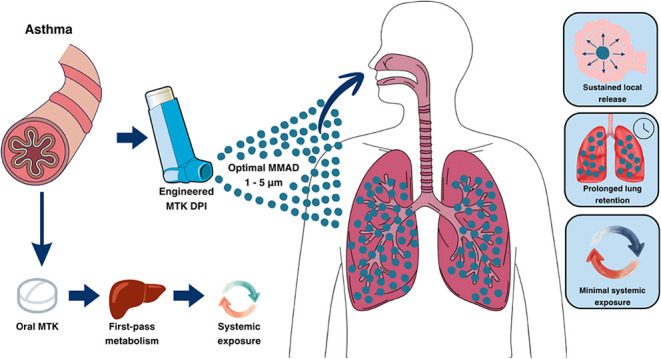

## 1 Introduction

Asthma is an important chronic inflammatory disease, which affects approximately 300 million people worldwide.^1^ The number of cases has increased in many countries, representing high costs, mainly related to its debilitating condition.^1^ The disease is characterized by inflammation and hyperresponsiveness of the airways, resulting in airflow obstruction caused by bronchoconstriction, wall edema, and mucus hypersecretion, which manifest as symptoms such as shortness of breath or difficulty breathing, persistent cough, wheezing, and tightness in the chest^2^ (Figure 1). Symptoms can be triggered by factors such as infections, the presence of allergens, occupational exposures, medications, exercise, and atmospheric pollutants.^3^ Asthma treatment involves maintaining control of symptoms and preventing their progression, avoiding the occurrence of exacerbation, instability, accelerated loss of lung function, and adverse effects.^1,4^ Therapeutic agents target inflammation and bronchoconstriction. Therefore, the first line of asthma management includes inhaled corticosteroids (ICS) and β2 agonists. ICS are considered the gold standard for treating the disease, as they can suppress multiple inflammatory processes simultaneously.^5^ Despite being potent anti-inflammatory agents, ICS have nonspecific action and are associated with important adverse effects, especially in long-term treatment, such as growth retardation in children, osteoporosis, reduction in bone mass, cataracts and glaucoma, adrenal suppression, and oropharyngeal diseases.^6^ ICS can be associated with long-acting β2-agonists (LABA) when treatment with ICS alone is not sufficient to maintain symptom control.^7^ However, during acute asthma exacerbations, short-acting β2-agonists (SABAs), such as salbutamol, can promote rapid relaxation of airway smooth muscles.^8^

**Figure 1:**
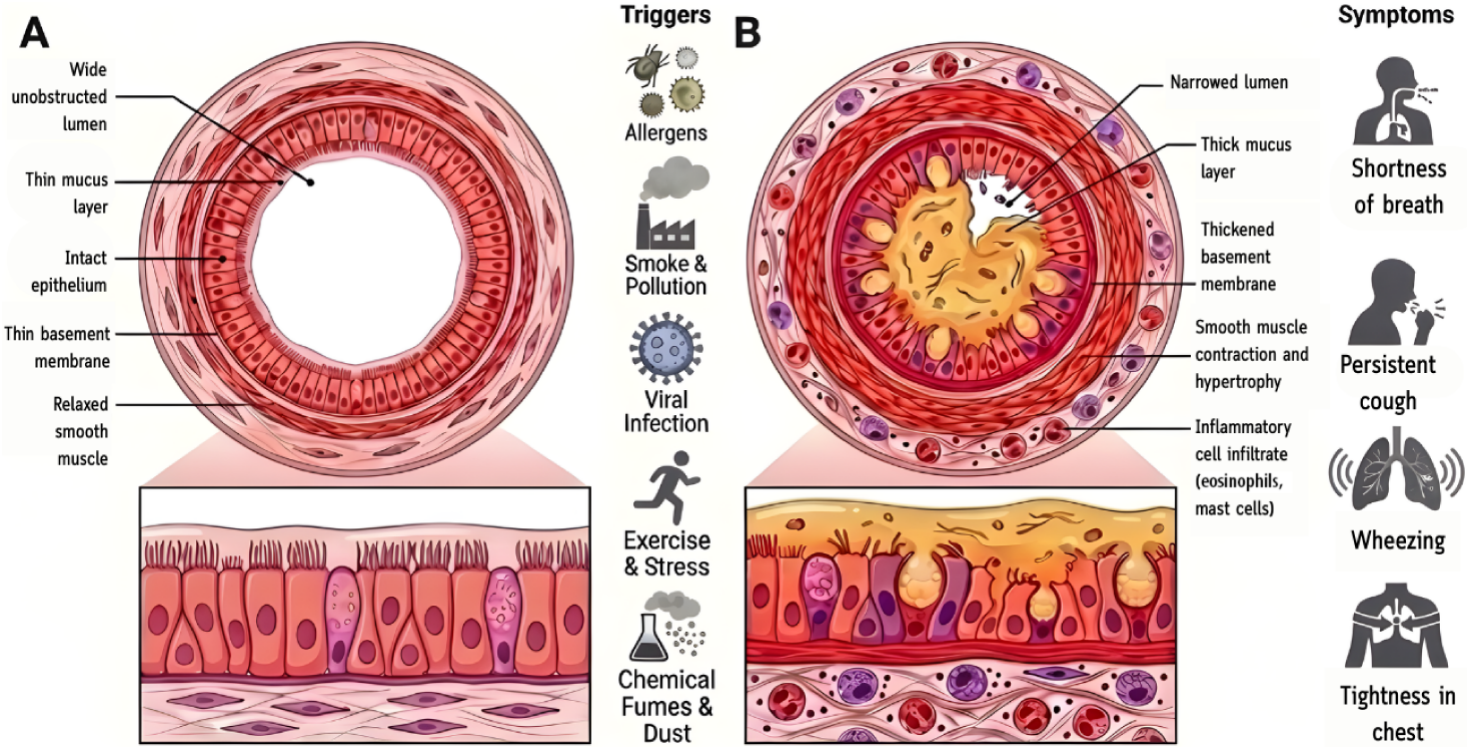
Structural and cellular features of a normal airway compared with an asthmatic airway, with common triggers and clinical manifestations. (A) Schematic cross-section of a nonasthmatic airway showing a wide, unobstructed lumen, a thin mucus layer, intact epithelium, thin basement membrane, and relaxed airway smooth muscle. The lower inset illustrates the corresponding epithelial lining with preserved architecture. (B) Schematic cross-section of an asthmatic airway showing characteristic airway remodeling and obstruction, including a narrowed lumen, thick mucus accumulation, thickened basement membrane, smooth muscle contraction and hypertrophy, and inflammatory cell infiltration, particularly eosinophils and mast cells. The lower inset highlights epithelial alterations associated with asthma, including mucus hypersecretion and inflammatory involvement of the subepithelial region. The central column summarizes representative asthma triggers, including allergens, smoke and pollution, viral infection, exercise and stress, and chemical fumes and dust. The right column depicts common symptoms associated with these airway changes, namely shortness of breath, persistent cough, wheezing, and chest tightness. This image was created with Google AI Studio using the Nano Banana Pro model.

Leukotriene receptor antagonists, such as montelukast (MTK), zafirlukast, ritolukast, and verlukast, are used as second-line adjunct therapies in patients with uncontrolled asthma.^9^ Cysteinyl leukotrienes (LTC_4_, LTD_4_, and LTE_4_) are potent inflammatory molecules that play a central role in asthma pathophysiology by activating cysteinyl leukotriene receptors (CysLT_1_, CysLT_2_, and CysLT_3_).^9,10^

Among these receptors, CysLT_1_ is the primary mediator of leukotriene-induced bronchoconstriction and airway inflammation in asthma. MTK acts as a potent and highly selective antagonist of CysLT_1_, thereby inhibiting key asthmatic responses such as airway smooth muscle contraction, mucus hypersecretion, increased vascular permeability, and eosinophil recruitment. CysLT_1_ receptors are abundantly expressed in airway smooth muscle and inflammatory cells, including eosinophils, lymphocytes, and macrophages, and are widely distributed throughout the bronchial and alveolar tissues in patients with asthma.^11–13^

MTK is indicated in adult and pediatric patients (from 2 years of age) and has demonstrated clinical efficacy for the prophylaxis and chronic treatment of asthma, including the prevention of daytime and nighttime symptoms, prevention of exercise-induced bronchoconstriction, improved lung function, and the treatment of patients with asthma sensitive to aspirin and in relieving allergic rhinitis symptoms. It may be administered as monotherapy or in combination with other agents, primarily ICS, providing additive benefits such as improved lung function, reduced symptoms and exacerbations, and a reduced need for rescue medication while also enabling ICS dose reduction without loss of clinical stability.^12,14,15^

MTK is currently marketed as a sodium salt in granules and in chewable or coated tablets. The recommended daily dosage is 10 mg tablet for adults and 5 mg chewable tablet for pediatric patients aged 6 to 14 years. MTK exhibits variable pharmacokinetics,^8,16^ characterized by moderate oral bioavailability (60–70%), extensive first-pass metabolism, a short elimination half-life, and wide peripheral tissue distribution,^16,17^ which is associated with important adverse effects, including neuropsychiatric events.^12^ Although these adverse effects do not invariably result in treatment discontinuation, their occurrence underscores the need for a careful risk–benefit assessment prior to prescribing MTK, as well as thorough counseling of patients and caregivers regarding the potential for neuropsychiatric events.^1^

Inhaled administration may deposit a substantial drug fraction in the lungs for systemic or local effect via a noninvasive route.^18^ The nature of the effect is driven by the rate and extent of drug absorption as well as its residence time in the lungs and the mechanisms of clearance.^19^ For locally acting inhaled drugs, maximizing unbound pulmonary concentrations while minimizing systemic exposure is essential to optimizing efficacy and safety. The inhalation route is a strategy for delivering locally acting drugs with limited oral bioavailability, such as MTK. In addition, pulmonary drug delivery can support faster onset of action when the therapeutic target is located in the airways while avoiding hepatic first-pass metabolism and minimizing systemic side effects.^20,21^ For some drugs targeting pulmonary diseases, inhaled administration has been associated with dose reductions of approximately 10-fold compared to oral dosing, although such dose-sparing has not yet been demonstrated for MTK in humans.^22^

Despite being a promising candidate for inhaled administration, no MTK product has yet been commercialized, and only a limited number of studies have evaluated its efficacy and safety via this route, although clinical studies have already reported its therapeutic effectiveness when administered by inhalation.^23,24^ This review examines current advances in the formulation of MTK for pulmonary administration, highlighting the main challenges and recent technological developments associated with this strategy. Emphasis is placed on particle engineering approaches, micro- and nanostructured delivery systems, excipient selection, and the role of inhalation devices in determining therapeutic efficiency. This review aims to provide a framework to guide the rational development of inhalable MTK systems and to support future clinical and industrial advances.

## 2 An Overview of Pulmonary Drug Delivery System

Understanding the structural complexity of the human respiratory system provides a unique and highly efficient environment for both local and systemic drug administration, offering significant advantages over the oral and intravenous routes. The lungs provide a large absorptive surface area of up to 100 m^2^, supported by extensive vascularization, relatively low enzymatic activity compared to the gastrointestinal tract and liver, and the avoidance of hepatic first-pass metabolism, which together make them an attractive route for drug delivery.^25–27^

The airway epithelium is the primary barrier to systemic absorption, and its composition varies along the respiratory tract.^28^ The conducting zone consists of a pseudostratified columnar layer with basal, goblet, and ciliated cells, covered by a pulmonary lining fluid composed of a periciliary sol layer and a mucus gel rich in mucins, proteins, lipids, and salts, which limits drug diffusion and promotes mucociliary clearance.^29,30^ Fast-dissolving drugs become available for pulmonary absorption and can reach the systemic circulation before clearance mechanisms (Figure 2). On the other hand, slowly dissolving or larger particles are transported toward the pharynx, with particles larger than 6 μm being cleared more readily (Figure 2).^25,30^

**Figure 2:**
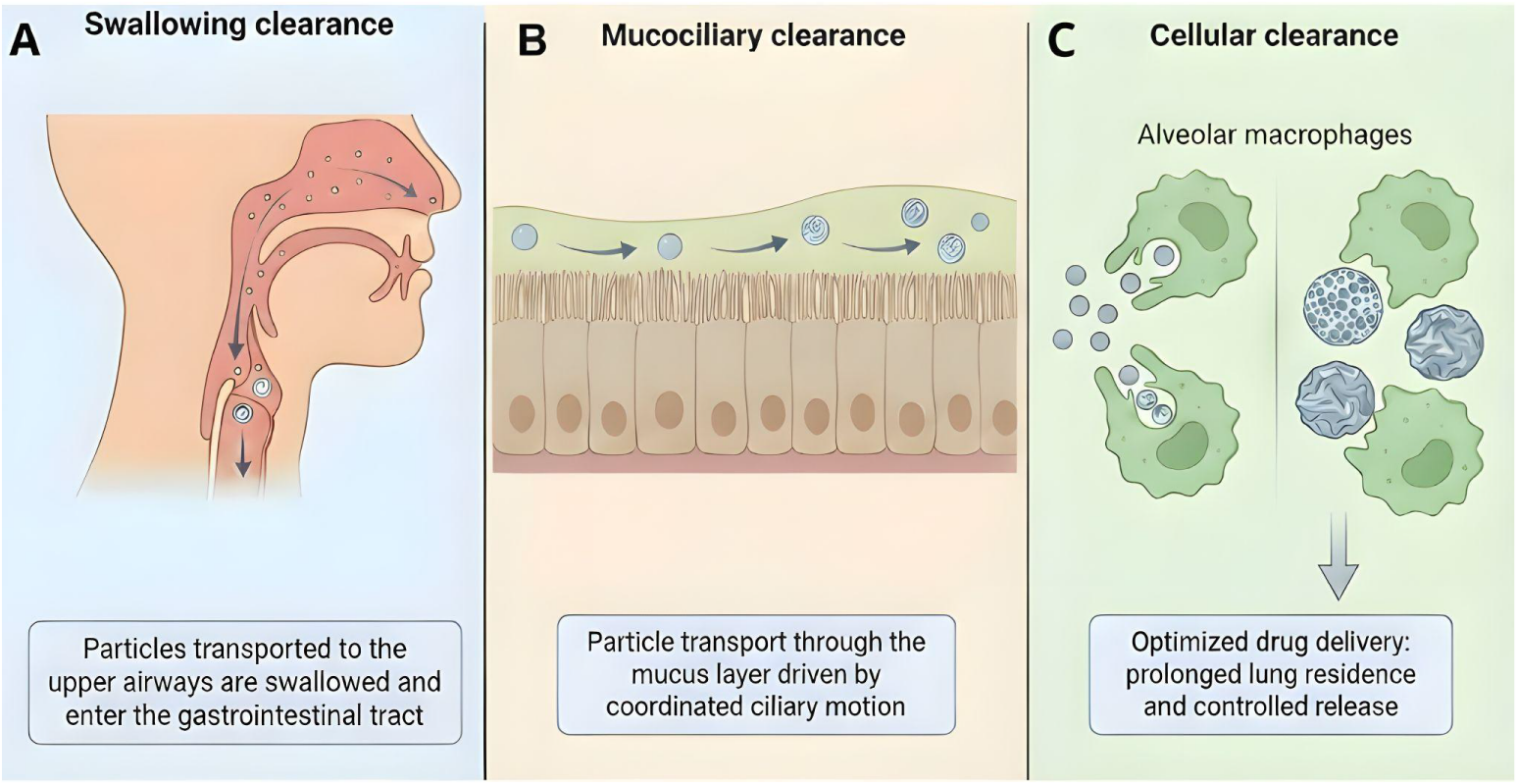
Schematic representation of the main pulmonary clearance mechanisms affecting inhaled particles after deposition in the respiratory tract. (A) Particles reaching the upper airways may subsequently be swallowed and enter the gastrointestinal tract. (B) In the conducting airways, particles entrapped in the mucus layer are transported toward the upper respiratory tract by coordinated ciliary beating. (C) In the alveolar region, particles may be recognized and internalized by alveolar macrophages, contributing to clearance from the deep lung. Reducing macrophage-mediated removal is therefore an important consideration in the design of pulmonary delivery systems, including montelukast (MTK)-containing formulations, as this may favor prolonged pulmonary residence and controlled drug release. Together, these mechanisms are major determinants of the retention and elimination of inhaled formulations. This image was created with Google AI Studio using the Nano Banana Pro model.

In the alveolar region, the epithelial barrier is significantly thinner, reaching about 0.1 μm, composed of type 1 cells, which cover 90% of the surface area, and type 2 cells. Here the mucus layer is replaced by a surfactant film (phospholipids, cholesterol, and proteins) that supports gas exchange and facilitates the diffusion and absorption of inhaled drugs.^31^ In addition, nonabsorptive clearance is carried out mainly by alveolar macrophages, which phagocytose insoluble particles in the 0.5–3 μm size range^25^ (Figure 2).

Once inhaled, the bioavailability of drugs in the lungs is influenced by solubility, dissolution rate, and epithelial permeability,^21^ while deposition depends on the aerodynamic behavior of inhaled particles, which determines the delivered dose and its regional distribution. The fate of the inhaled drug is determined by three mechanisms: 1) inertial impaction: larger particles >5 μm inhaled at high flow rates tend to deposit in the upper airways and are often swallowed;^32,33^ 2) gravitational sedimentation: particles <1 μm are often exhaled due to low sedimentation velocity, while those in the 1–5 μm range deposit in bronchi, bronchioles, and alveoli as airflow decreases;^34–37^ 3) diffusion: ultrafine particles <1 μm undergo Brownian motion and may deposit in the alveoli if not exhaled.^20,33^

Most inhaled drugs require delivery devices such as nebulizers, pressurized metered-dose inhalers (pMDIs), and dry powder inhalers (DPIs).^38^ These systems administer medications in aerosol form, which consists of droplets or solid particles suspended in air or in a pressurized gaseous medium.^39^ Device selection should consider patient-, drug-, and device-related factors, including cost, maintenance, and ease of use.

*In vitro* characterization is a critical step in evaluating inhalation formulations. Particle size, morphology, and density determine the aerodynamic behavior and deposition within the respiratory tract. Aerosolization performance is typically assessed using cascade impactors, such as the Next Generation Impactor (NGI) and Andersen Cascade Impactor (ACI), which quantify the aerodynamic particle size distribution across stages representing different lung regions. Key performance parameters for inhalation products include the mass median aerodynamic diameter (MMAD), geometric standard deviation (GSD), fine particle fraction (FPF), and emitted dose (ED). The MMAD corresponds to the aerodynamic diameter at which 50% of the drug mass is deposited, with values within the 1–5 μm range generally considered optimal for reaching the deeper lung regions.^40^ The GSD reflects the particle size variability, while FPF indicates the proportion of respirable particles. The ED reflects the fraction of the nominal dose that is effectively released from the inhaler and thus available for pulmonary deposition. Inhaled formulations must also exhibit adequate physicochemical stability and minimal drug retention in the device.^41^

## 3 Inhaled MTK Strategies

Considering the potential advantages of pulmonary delivery for MTK, a literature search was conducted to identify inhaled formulations of the drug. The bibliographic review revealed that, despite the widespread oral use of MTK, its pulmonary administration has been minimally investigated. Twenty articles were identified, including 16 on DPIs, one on nebulized montelukast, one on pMDIs, and two articles reporting results related to clinical studies of inhaled montelukast. The full search strategy, eligibility criteria, and stepwise study selection counts are detailed in Section 8.

The identified studies were analyzed according to the formulation strategy, particle engineering approach, inhalation device, and reported outcomes. The limited number of investigations on inhaled MTK formulations underscores a translational gap between preclinical formulation development and clinical application, which may contribute to the absence of approved pulmonary MTK products in the pharmaceutical market.

The following sections illustrate the proposed delivery systems for inhaled administration of MTK using particle engineering techniques applicable to different inhalation devices (Figure 3).

**Figure 3:**
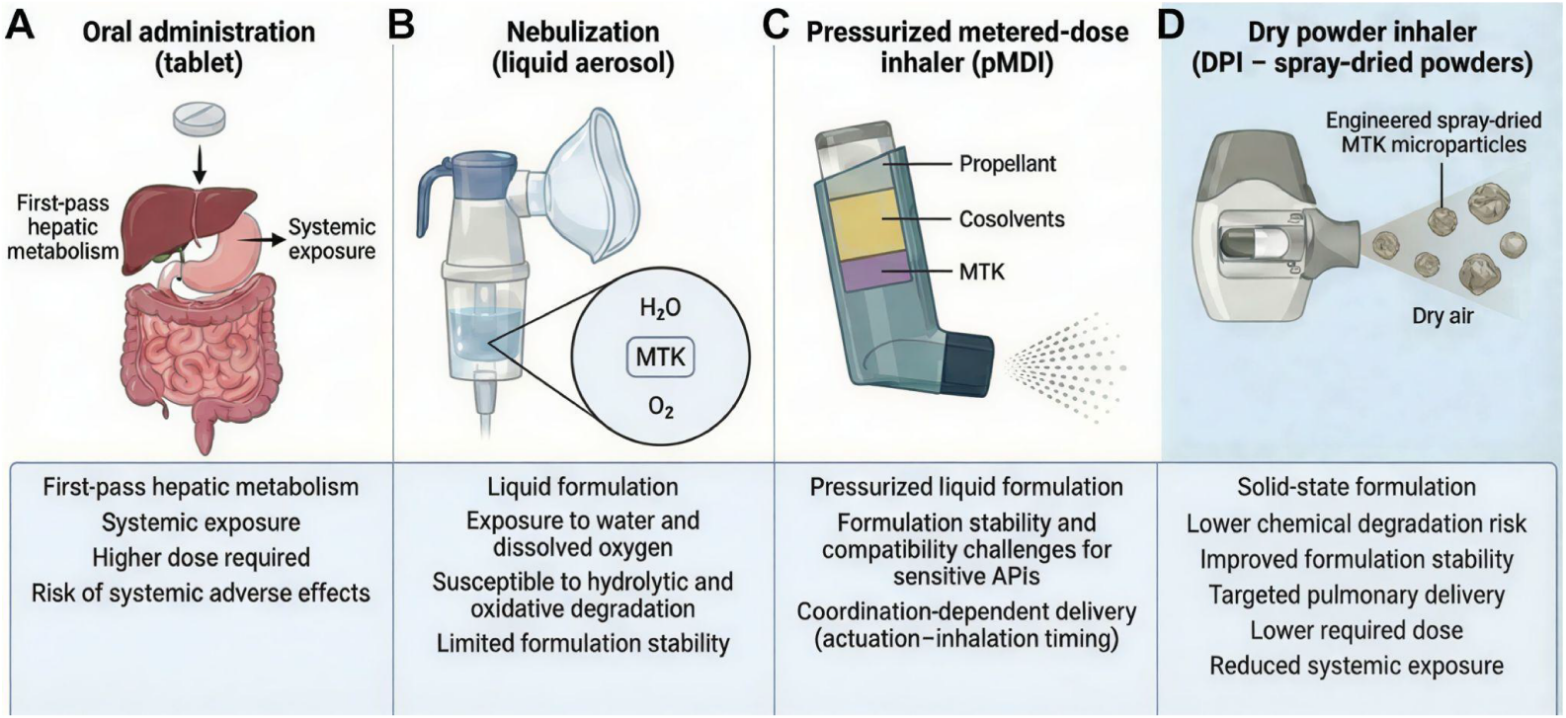
Overview of oral and pulmonary delivery approaches for montelukast (MTK), illustrating the principal limitations of conventional administration routes and the potential advantages of dry powder inhalation. (A) Oral administration exposes MTK to gastrointestinal absorption followed by first-pass hepatic metabolism, resulting in systemic exposure and generally necessitating higher doses, with a consequent increase in the risk of systemic adverse effects. (B) Nebulized formulations deliver MTK as a liquid aerosol, but the presence of water and dissolved oxygen may compromise chemical stability through hydrolytic and oxidative degradation. (C) Pressurized metered-dose inhalers (pMDIs) incorporate MTK into a pressurized liquid formulation containing propellant and cosolvents; however, this format may impose formulation stability and compatibility constraints for sensitive active pharmaceutical ingredients and depends on appropriate actuation–inhalation coordination. (D) By contrast, dry powder inhalers (DPIs) administer engineered spray-dried MTK microparticles in a solid-state format using inspiratory airflow, an approach that may improve formulation stability, reduce degradation risk, enable more selective pulmonary delivery, lower dose requirements, and reduce systemic exposure. This image was created with Google AI Studio using the Nano Banana Pro model.

### 3.1 Nebulizers

Nebulizers, which may be jet, mesh, or ultrasonic type, deliver medications in liquid form as a mist. They allow the administration of drug mixtures and large doses, can be used with normal tidal breathing, and are suitable for patients of all ages, including unconscious individuals.^42^ Despite their widespread use in emergency settings and hospitalizations, particularly during severe asthma attacks, nebulizers have some drawbacks: aerosolization requires extended time, the devices are not very portable, they rely on an external power source, are relatively costly, and demand frequent disassembly and cleaning after each use.^43,44^

Only one study has reported the use of nebulization as an inhaled delivery strategy for the MTK. Muraki et al. investigated the effect of nebulized MTK sodium on airway hyperresponsiveness in an animal model of asthma, using cysteinyl leukotriene-induced (LTC_4_ or LTD_4_) bronchoconstriction as a stimulus. Airway response was assessed by changes in peak pressure of airway opening (P_ao_) in animals pretreated with a 15 min inhalation of either 10 mg/mL MTK or saline. In saline-treated controls, P_ao_ increased rapidly after leukotriene inhalation, reaching maximal levels within 30 s to 5 min. In contrast, nebulized MTK markedly suppressed leukotriene-induced increases in P_ao_, reducing the responses to baseline levels. Pretreatment with inhaled MTK effectively inhibited the peak P_ao_ elevations induced by both inhaled and intravenous LTC_4_ and LTD_4_.^45^

### 3.2 Pressurized Metered-Dose Inhalers

pMDIs are the most commonly used inhalation devices.^25^ These systems aerosolize drug solutions or suspensions using a propellant gas, delivering medication directly to the lungs. They offer fast, portable, and consistent drug delivery, particularly when compared with nebulizers.^46^ However, effective use often requires coordination between device actuation and patient inhalation, which can limit their use.^44^

Recent developments include breath-actuated pMDIs, such as Easibreathe, which automatically releases the dose upon detection of the patient’s inhalation.^43^ In the 1950s, pMDIs used chlorofluorocarbons (CFCs) as propellants. Over time, due to environmental concerns, these were replaced by hydrofluoroalkane (HFA) propellants, which are inert, effective, nontoxic, and safe. HFAs are rapidly absorbed, eliminated by the lungs, and do not contribute to ozone layer depletion. However, high-pressure HFA inhalers are not suitable for all drug types, particularly biologics, due to limitations in the deliverable dose range and issues related to physicochemical instability (e.g., freezing or precipitation).^25,38^

Using a three-level factorial design with two factors, Sawatdee et al. proposed 22 different pMDI formulations for MTK pulmonary delivery as an alternative to oral administration. The formulations included MTK as the active ingredient, HFA-134a or mixed HFA-134a/HFA-227 propellants, with ethanol as a cosolvent and polyethylene glycol (PEG) 400 as a solubilizer. Eight formulations met the drug delivery dose criteria of 80–120% of the labeled claim (15 μg of MTK per puff). Among these, all demonstrated favorable aerosol properties, with FPF exceeding 42% and MMAD within the optimal 1–5 μm range. Notably, the formulation containing 15% v/v ethanol, 5% v/v PEG 400, and HFA-134a propellant exhibited the highest FPF at 58.8% and an optimal MMAD of 2.0 μm. *In vitro* cytotoxicity assays using three lung cell lines (A549, NR8383, and Calu-3) demonstrated that all formulations maintained cell viability over 90%, indicating negligible cytotoxicity. Furthermore, the formulations did not induce significant production of inflammatory mediators (nitric oxide, TNF-α, and IL-1β) in alveolar macrophages, suggesting a low potential for pulmonary irritation or inflammation.^47^

### 3.3 Dry Powder Inhalers

As an alternative to pMDIs, DPIs were introduced in the 1960s. DPIs are portable, breath-actuated devices that do not require propellants and deliver drugs in dry powder form through shear-induced aerosolization.^48^ These devices are often preferred because they eliminate the need for coordination between device actuation and inhalation. However, the delivered dose is highly dependent on the patient’s inspiratory flow rate, which may lead to variability in pulmonary drug deposition.^42^

DPI can be classified into two main formulation approaches: carrier-based systems and carrier-free systems.^49^ In conventional carrier-based formulations, micronized drug particles (1–5 μm) are blended with a larger carrier excipient (50–200 μm), typically lactose.^50^ The carrier improves powder flowability, facilitates drug release from the inhaler, and enhances pulmonary deposition.^51^ It also increases the total powder mass, contributing to reduced dose variability.^52^ In contrast, carrier-free systems are composed solely of drug particles or drug-excipient composite particles and are designed to overcome issues associated with conventional formulations, such as poor blend uniformity, particle cohesiveness, and limited dosing capacity.^53^ Through particle engineering techniques, powders with appropriate size and improved aerodynamic properties can be produced, thereby optimizing lung deposition.^18^ DPI formulation methods include spray drying (SD),^48,54–56^ spray freeze-drying,^57^ electrospraying,^58^ solvent evaporation method,^17,59–61^ jet milling,^62^ and supercritical fluid technology.^63^

The identified studies indicate that MTK DPI formulation strategies were consistently focused on achieving respirable particle size, adequate aerodynamic performance, and reproducible aerosolization behavior required for the DPI administration. As a result, the reported MTK DPI formulations generally achieved an MMAD within the respirable range and FPF suitable for pulmonary deposition, demonstrating the technical feasibility of MTK administration via DPIs.^54,56,58,59,64^

Among the particle engineering techniques applied, SD emerged as the predominant method for the MTK DPI development. This technique was primarily selected due to its ability to produce inhalable particles or composite systems with controlled size, morphology, and solid-state properties in a single, reproducible, and scalable process.^65^ Several studies reported that variations in formulation composition and SD conditions significantly affected particle formation and aerosolization behavior, underscoring the importance of process control in the development of MTK DPI systems.^48,54,56^ Although other methods for obtaining MTK particles suitable for DPI formulations have been reported, their application remains comparatively limited.^17,58,59^

Nevertheless, most investigations employing particle engineering approaches remain restricted to *in vitro* characterization^48,54–58,61,66^ or preclinical evaluation,^17,64,67^ indicating that further studies addressing *in vivo* pulmonary performance and clinical translation are required.

#### 3.3.1 Excipients

Although the physicochemical properties of powders can be modulated by SD, the production of DPIs with an appropriate aerodynamic performance remains challenging. Consequently, many DPI formulations combine the active pharmaceutical ingredient (API) with excipients to produce composite particles that enable efficient device delivery and pulmonary deposition.^68^ Excipients are commonly incorporated into DPI formulations to modify bulk powder properties, improve physical and chemical stability, and modulate the pharmacokinetic and pharmacodynamic behaviors of the drug.^69^ This aspect is particularly relevant for MTK, a hygroscopic drug, for which the concentration within the formulation markedly influences powder behavior.^48^ Previous SD studies using the pure API have shown that MTK powders exhibit poor physical stability and a pronounced tendency toward irreversible agglomeration after processing.^48,56^ Therefore, excipient selection plays a critical role in the development of inhalable MTK formulations to ensure a consistent aerosol performance and therapeutic efficacy. The potential impact of an excipient on product quality depends not only on its intrinsic physicochemical properties but also on its concentration within the formulation.

From a regulatory perspective, excipients intended for pulmonary delivery should ideally present a well-established history of use and a characterized safety profile. In this context, lactose is currently the only excipient included in the inactive ingredient list of the FDA for inhalation use,^70^ primarily employed as a carrier in marketed DPI products. The use of alternative or nontraditional excipients requires additional safety and toxicological evaluation to support their suitability for inhalation.

Sugars, polymers, and amino acids represent the main classes of excipients employed in DPI formulations, with particular emphasis on those reported for inhalable MTK systems, each contributing distinct functionalities to the powder performance and stability. Sugars play a central role, primarily acting as diluents and flow-enhancing agents that enable accurate dosing of micronized APIs while also serving as stabilizers in SD systems.^41,71^ Lactose is the most commonly used linear sugar in carrier-based DPI formulations because of its safety, widespread availability, and compatibility with low-molecular-weight drugs.^64^ It is commonly produced by SD, milling, or sieving, allowing access to materials with tailored physicochemical properties.^72^ Lactose also improves powder flowability, as demonstrated by enhanced performance with increasing concentrations in SD systems.^64^ However, its application as a stabilizer is constrained by its tendency to form amorphous, hygroscopic solids as well as by its reducing nature, which may promote Maillard reactions and compromise stability.^73–75^ Alternatively, mannitol, another linear sugar, has emerged as an excipient with advantageous properties, including low hygroscopicity, nonreducing character, and high crystallinity, which improve powder stability and flowability.^76^ It also enhances the SD yield and reduces particle agglomeration.^48,56^ Despite being generally recognized as safe, high doses may induce hyperosmolar airway conditions and should be carefully considered in the formulation design.^41,77^

Cyclodextrins (CDs) are cyclic sugars with a hydrophilic exterior and a lipophilic cavity that can form inclusion complexes with poorly soluble drugs, thereby enhancing solubility and stability.^78,79^ In addition to improving solubility, CDs can protect APIs from degradation, mask undesirable properties, and modulate drug release.^80–82^ In DPI formulations, CDs have demonstrated superior performance compared to linear sugars, producing spherical, porous particles with reduced aggregation and improved aerodynamic behavior (PPF > 80%).^57^

Polymers, both natural and synthetic, offer high versatility due to their tunable physicochemical properties, which directly influence particle characteristics, drug release, and therapeutic performance.^29,83^ Natural polymers such as polysaccharides (e.g., alginate, chitosan) and proteins (e.g., albumin, collagen) are typically biodegradable and biocompatible but may present challenges related to variability and scalability.^73^ Chitosan is particularly attractive due to its mucoadhesive properties and favorable safety profile, enabling the production of inhalable microparticles with high encapsulation efficiency and tunable properties depending on polymer concentration.^55^ Synthetic polymers such as polylactic acid (PLA), poly­(lactic-*co*-glycolic) acid (PLGA), poly-ε-caprolactone (PCL), and poly­(vinyl alcohol) (PVA) provide greater reproducibility and control over formulation attributes.^84^ Among these, PLGA is widely used due to its adjustable degradation and drug release profiles, which can be modulated by the lactide-to-glycolide ratio and molecular weight.^29,85^ Faster drug release is associated with higher glycolide content due to increased hydrophilicity and degradation rate, while more hydrophobic compositions enable sustained release.^60^

Amino acids have gained attention as functional excipients, particularly for improving aerosol performance.^84^ Among them, l-leucine is the most extensively studied because of its low solubility and surface activity, which promote surface enrichment during SD.^86,87^ This leads to the formation of a hydrophobic crystalline shell, resulting in corrugated, low-density particles with enhanced dispersibility and reduced interparticle cohesion.^73,88^ Consequently, l-leucine-containing DPI formulations exhibit improved flowability, high FPF, and optimal aerodynamic diameters.^56^ Its role as both a dispersibility enhancer and stabilizer has been consistently demonstrated, contributing to high production yields, efficient deagglomeration, and improved resistance to moisture-induced instability.^48,56,89^

#### 3.3.2 MTK Inhalable Particles

##### 3.3.2.1 Microparticulate Systems

Microparticulate drug delivery systems have gained relevance in the pharmaceutical field due to their capacity to overcome important formulation challenges, including the delivery of poorly soluble drugs, enabling controlled or sustained release over extended periods, and preserving the stability of active compounds.^90^ In the context of pulmonary delivery, microparticles have been widely investigated as DPI formulations due to their ability to modulate aerodynamic behavior and drug release while enhancing lung deposition. Table 1 presents the MTK inhalable microsystems described in the literature.

**Table 1:** Montelukast (MTK) Inhalable Microsystems: Production Methods, Formulation Composition, Aerosol Performance, and Physicochemical Characteristics^a^

production method	excipients	formulation	device	shape	particle size (μm)	MMAD (μm)	GSD (μm)	FPF (%)	density (g/cm^3^)	zeta potential (mV)	dissolution	ref
Spray drying	Chitosan (polymer)	Microspheres	DPI	NR	7–12	NR	NR	NR	NR	NR	Burst drug release (50–60%) within 30 min and sustained release for 8 h (90%)	^55^
	Glutaraldehyde (cross-linking agent)											
Spray drying	2% Xyloglucan (polymer)	Microspheres	DPI (Rotahaler)	Spherical morphology with smooth surface	0.9–6	2.53 (60 L/min)	1.74	43.8	NR	–18.2	139 min for 90% drug dissolution	^64^
	1% Lactose monohydrate											
W1/O/W2 Double-emulsion–solvent-evaporation and lyophilization method	3% PEI (polyethylenimine—porosigen)	Large porous polymeric particulate system	DPI (HandiHaler)	Near-perfect spheres with porous surface	7.72	2.51 (28.3 L/min)	NR	NR	0.10	+7.61	Burst drug release (∼6–7%) within 30 min and sustained release for 4 days (31%)	^59^
	1% w/v PVA											
	Polymer (PLA or PLGA)											
Spray drying	Mannitol (matrix former), ovalbumin (particle shaper), and l-leucine (dispersing aid)	Microparticles	DPI (Aerolizer)	NR	2.26 (DM)	0.59 (60 L/min DM)	NR	8.87 (DM)	0.59 (DM)	NR	More than 95% drug dissolution within 15 min	^56^
	DM—50% MTK + 50% mannitol				3.17 (DL)	1.73 (60 L/min DL)		60.55 (DL)	0.18 (DL)			
	DL—50% MTK + 50% l-leucine				2.53 (DML)	1.36 (60 L/min DML)		52.31 (DML)	0.38 (DML)			
	DML—50% MTK + 25% l-leucine + 25% mannitol											
Spray drying	Ammonium bicarbonate (ABC)	Microparticles	DPI (Aerolizer)	Spherical, smooth without aggregated particles	NR	3.63 (60 L/min)	1.86	48.3	NR	NR	NR	^54^
O/W emulsion–solvent-evaporation and lyophilization method	Eudragit RS100 (polymer) and Inhalable lactose carrier (DPI)	Microspheres	DPI	Spherical, firm, smooth particles	4.92 (A)	2.22 (A)	NR	NR	NR	NR	Sustained release with no less than 74% within 12 h	^61^
					4.72 (B)	2.72 (B)						
	Drug:polymer ratios of 1:1 (A); 1:2 (B); 1:3 (C); and 1:4 (D)				4.69 (C)	2.67 (C)						
					4.59 (D)	2.92 (D)						
W1/O/W2 Double-emulsion–solvent-evaporation and lyophilization method	3% PEI (polyethylenimine—porosigen)	Dual-drug (MTK and LMWH) polymeric particulate system	DPI (HandiHaler)	Spherical in shape with numerous pores on the surface	4.03 (A)	1.95 (A)	NR	61.43 (D)	0.22 (A)	–16.5 (A)	Burst drug release within 30 min and sustained release for 4 days	^60^
	1% w/v PVA				5.07(B)	2.99 (B)			0.27 (B)	–20.7 (B)		
	Polymers: PLA (A) or PLGA 85:15 (B) or PLGA 50:50 (C) or PEG–PLGA (D)				7.51 (C)	3.66 (C)			0.23 (C)	–26.3 (C)		
					10.34 (D)	4.14 (D) (28.3 L/min)			0.16 (D)	–5.03 (D)		
W1/O/W2 Double-emulsion–solvent-evaporation and lyophilization method	3% PEI (polyethylenimine—porosigen)	MTK encapsulated PLGA-based particles	DPI (HandiHaler)	Spherical with porous surface	∼7–8 (PEI-1)	∼2.5 (PEI-1)	NR	NR	∼0.10 (PEI-1)	∼+7.5 (PEI-1)	Burst drug release (∼5–7%) within 30 min and sustained release for 4 days (∼30%)	^17^
	1% w/v PVA				∼4–5 (PEI-2)	∼2.5 (PEI-2)			∼0.25 (PEI-2)	∼+7.6 (PEI-2)		
	Polymers: PLA (PEI-1) or PLGA 85:15 (PEI-2) or PLGA 50:50 (PEI-3)				∼4 (PEI-3)	∼1.8 (PEI-3) (28.3 L/min)			∼0.20 (PEI-3)	∼+10 (PEI-3)		
Spray drying	40% Mannitol (M2) or 40% ovalbumin (O2) as matrix formers, 5% Lecithin (as binder) and 50% l-leucine (as dispersing agent)	Modified soft agglomerate composite microparticles	DPI (Aerolizer)	Spherical, agglomerated though nonfused particles showing rough surfaces	4.74 (M2)	1.68 (M2)	NR	NR	0.322 (M2)	NR	More than 95% drug dissolution within 5 min	^48^
					4.75 (O2)	1.31 (O2) (60 L/min)			0.201 (O2)			
Electrospraying	Dichloromethane (organic solvent)	Dual-drug (MTK and budesonide) particulate system	DPI (Aerolizer)	Spherical and flattened with smoother surface morphology	5.93	3.68 (60 L/min)	1.83	38.65	NR	NR	More than 60% drug dissolution within 3 min and more than 80% in 15 min	^58^
	Inhalation grade lactose (carrier)											
Spray freeze-drying	Erythritol, d-Lactose Monohydrate, d-Mannose, d-Trehalose dihydrate, Raffinose pentahydrate (sugar-based carrier)	Microparticles	DPI (Cyclohaler)	Irregular, nonspherical and aggregated (Sugar-based particles)	5–12 (CD particles)	NR	NR	>40	NR	NR	NR	^57^
	α-CD, β-CD, γ-CD, HP-β-CD, HBCD			Spherical and porous (CD particles)								

a NR = not reported, DPI = dry powder inhaler, MMAD = mass median aerodynamic diameter, GSD = geometric standard deviation, and FPF = fine particle fraction.

Among these systems, polymer-based microparticles have emerged as a particularly promising strategy for the pulmonary delivery of MTK. Their high physicochemical stability, substantial drug-loading capacity, relatively low drug release rate, and prolonged pharmacological activity make them attractive carriers for inhalation therapies.^91^ Mahajan and Gundare developed MTK-loaded microspheres by SD using xyloglucan as a polymeric matrix and lactose as a carrier. The optimized formulation, containing 2% (w/v) xyloglucan and 1% (w/v) lactose, exhibited suitable aerodynamic properties when evaluated using an ACI. The microspheres achieved an MMAD of 2.53 μm, a GSD of 1.74, and an FPF of 43.8%, indicating efficient deposition in the deep lung regions. Additionally, more than 89% of the nominal dose was emitted from the inhaler, demonstrating excellent powder dispersibility and inhalation performance.^64^ Satishkumar et al. prepared MTK polymeric microparticles with different drug-to-polymer ratios using solvent evaporation, then blended them with lactose to create a DPI system. Four formulations were proposed using MTK sodium and synthetic polymer Eudragit RS100 at ratios of 1:1, 1:2, 1:3, and 1:4. Microparticles were prepared via oil-in-water emulsification and solvent evaporation, blended with Lactohale LH100 as a carrier, and encapsulated into size-3 gelatin capsules with a fill weight of 20 mg. Drug content uniformity ranged from 90% to 110% of the theoretical value. The resulting microparticles were spherical with smooth surfaces and with mean diameters between 4.59 and 4.92 μm. ACI analysis at 60 L/min revealed that the 1:1 formulation achieved an emitted dose of 91.6% and a respiratory fraction of 38.4%, reflecting efficient deposition in the lower respiratory stages (stages 2–8 of the impactor). An MMAD of 2.22 μm further confirmed its suitability for deep lung delivery and potential therapeutic efficacy.^61^

While conventional polymeric microparticles provide good control over drug release and lung deposition, large porous particles (LPPs) represent an advanced approach to further improve pulmonary delivery. LPPs are composed of low-density particles (typically below 0.4 g/cm^3^) with geometric diameters greater than 5 μm,^44,92^ resulting in a small aerodynamic diameter, enabling efficient deposition in the deep lung regions.^53^ The relatively large geometric size of LPPs also minimizes particle agglomeration and improves dispersion compared to conventional microparticles, thereby enhancing aerosol performance.^93,94^ Moreover, LPPs are considered a promising strategy for achieving prolonged therapeutic effects, as their large physical size helps them evade macrophage phagocytosis, while their low density allows them to behave aerodynamically like smaller particles, facilitating deep lung deposition and reducing mucociliary clearance.^85^

LPPs are commonly prepared using the double emulsion method, followed by solvent evaporation through SD or spray freeze-drying.^44^ Patel et al. conducted studies on MTK-containing large porous particles using the water/oil/water (W1/O/W2) double-emulsion solvent evaporation technique. The formation of the porous structure in LPPs generally requires pore-forming agents, or porogens, such as volatile liquids, ammonium carbonate, or ammonium bicarbonate.^53,94,95^ Polyethylenimine (PEI) was incorporated to produce highly porous particles, which were spherical with porous surfaces and exhibited an MMAD of 2.51 ± 0.12 μm, approximately half the volume-based mean diameter of 7.72 μm, reflecting their low tapped density of 0.10 g/cm^3^. The authors suggested that PEI increased the osmotic pressure of the internal aqueous phase, drawing water into the particle core via aqueous channels, which were subsequently removed by lyophilization, forming surface pores.^17,59^ Compared with smaller nonporous particles, the MTK large porous particles also exhibited reduced uptake by alveolar macrophages and achieved greater deposition within the lungs.^17^

Nevertheless, improvements in DPI powders’ performance are not exclusively dependent on particle porosity but may also be strongly influenced by processing parameters and formulation, such as solvent system. Faramarzi et al. demonstrated that the incorporation of ammonium bicarbonate as a pore-forming agent during SD failed to improve particle porosity or aerosol performance, providing no enhancement in FPF or MMAD and, instead, leading to a reduction in production yield. In contrast, a systematic evaluation of formulation variables revealed that by optimizing the solvent system to a 2:1 ethanol-to-water ratio, the authors obtained smooth, spherical MTK microparticles with minimal aggregation, achieving an FPF of 48.3% and an MMAD of 3.5 μm, which are considered appropriate for pulmonary deposition. Formulations based solely on ethanol resulted in highly aggregated particles with inferior deposition potential.^54^

##### 3.3.2.2 Nanostructured Systems

Nanostructured drug delivery systems, typically ranging from 1 to 1000 nm, include polymeric nanoparticles, polymer micelles, solid lipid nanoparticles (SLN), nanostructured lipid carriers (NLC), and liposomes.^96^ They have emerged as a promising platform for pulmonary drug delivery due to their ability to enhance the solubility of poorly water-soluble drugs, modulate drug release, prolong pulmonary residence time, and potentially reduce systemic exposure.^31,53,97^ However, because of their small size and strong cohesive forces, nanoparticles are typically formulated with micrometer-sized carriers or as microparticle agglomerates to achieve adequate aerosolization and pulmonary deposition following inhalation.^98^ Table 2 shows MTK inhaling nanosystems described in the literature.

**Table 2:** Montelukast (MTK) Inhalable Nanosystems: Production Methods, Formulation Composition, Aerosol Performance, and Physicochemical Characteristics MTK^a^

prod. method	excipients	formulation	device	shape	particle size (μm)	MMAD (μm)	GSD (μm)	FPF (%)	density (g/cm^3^)	zeta potential (mV)	dissolution	ref
Ionic gelation method	0.213% Chitosan (polymer)	Polymeric nanoparticles	DPI	NR	0.2225	NR	NR	NR	NR	+24.9	24% drug release within 30 min and sustained release for 24 h (70%)	^66^
	Tripolyphosphate (TPP) (cross-linking agent)											
	1.5 chitosan:TPP ratio											
Melt emulsification-ultrasonication method	Precirol ATO5 (solid lipid) and Capryol-90 (liquid lipid) in a 7:3 ratio	Lipid nanoparticles (nanostructured lipidic carriers; NLC)	DPI (Rotahaler)	NR	0.1846 (MNLC)	3.24 (30 L/min)	1.78 (30 L/min)	69.98 (30 L/min)	0.051	+37.7	15.35% drug release within 2 h and sustained release for more than 24 h (31%)	^67^
	1% Pyrrolidonecarboxylic acid salt of L-cocyl arginine ethyl ester (CAE) (surfactant)				15.1 (MNLC-DPI)	2.83 (60 L/min)	1.61 (60 L/min)	90.22 (60 L/min)				
	3% mannitol as carrier/cryoprotectant											
Hot homogeneization method	Compritol 888 ATO, Tween 80, and poloxamer 407	Solid lipid nanoparticles (SLN)	DPI (Easyhaler)	Regular and spheroidal shape DPI particles	<0.100 (SLN)	5.471 (spray-dried SLN DPI)	2.810 (spray-dried SLN DPI)	11.746 (spray-dried SLN DPI)	0.883 (spray-dried SLN DPI)	NR	∼14% drug release within 24 h	^99^
					1.098 (spray-dried SLN DPI)	5.892 (spray-dried SLN DPI with mannitol)	1.975 (spray-dried SLN DPI with mannitol)	16.178 (spray-dried SLN DPI with mannitol)	2.146 (spray-dried SLN DPI with mannitol)			
Spray drying	4% mannitol or lactose as carrier				1.25 (spray-dried SLN DPI with mannitol)	5.335 (spray-dried SLN DPI with lactose) (60 L/min)	2.068 (spray-dried SLN DPI with lactose) (60 L/min)	21.025 (spray-dried SLN DPI with lactose) (60 L/min)	2.128 (spray-dried SLN DPI with lactose)			
					1.68 (spray-dried SLN DPI with lactose)							
Ionic gelation method	∼40% Chitosan (polymer)	Polymeric nanoparticles physically admixed with lactose spray-dried microspheres	DPI (HandiHaler)	Irregular MTK nanoparticles accumulated on spherical spray-dried lactose microspheres	0.22–0.38 (nanoparticles)	1.23 (F2)	2.95 (F2)	28.45 (F2)	0.514	+22.23 (F2)	Burst drug release (∼20–40%) within 30 min and sustained release for 25 h (∼100%)	^100^
	∼30% Tripolyphosphate (TPP) (cross-linking agent) (F2)					2.34 (F4)	2.91 (F4)	64.55 (F4)		+18.06 (F4)		
Freeze-drying	0.025% Tween 80 (F4) or 10.2% hyaluronic acid (F5) or 10.2% l-leucine (F6)				5.6 (lactose microspheres)	1.78 (F5)	2.92 (F5)	63.92 (F5)		+14.1 (F5)		
	Lactose microspheres as carrier					1.34 (F6) (48 L/min)	4.78 (F6)	83.22 (F6)		+11.4 (F6)		
Ionic gelation method	∼40% Chitosan (polymer)	Polymeric nanoparticles embedded in lactose or mannitol microparticles	DPI (HandiHaler)	Triangular structure MTK nanoparticles	0.276 (nanoparticles)	1.078 (mannitol embedded nanoparticles)	3.603 (mannitol embedded nanoparticles)	25.05 (mannitol embedded nanoparticles)	0.508 (mannitol embedded nanoparticles)	+14.1	Burst drug release (∼30–40%) within 30 min and sustained release for 24 h (∼100%)	^101^
	∼28% Tripolyphosphate (TPP) (cross-linking agent)											
Freeze-drying	10.2% hyaluronic acid			Partially spherical lactose or mannitol nanoembedded microparticles	1.822 (mannitol embedded nanoparticles)	2.563 (lactose embedded nanoparticles) (60 L/min)	2.237 (lactose embedded nanoparticles)	31.37 (lactose embedded nanoparticles)	0.536 (lactose embedded nanoparticles)			
	Lactose or mannitol				2.352 (lactose embedded nanoparticles)							

a NR = not reported, DPI = dry powder inhaler, MMAD = mass median aerodynamic diameter, GSD = geometric standard deviation, and FPF = fine particle fraction.

In the context of MTK, several nanostructured approaches have been investigated to overcome its limited aqueous solubility and variable oral pharmacokinetics. Polymeric nanoparticles, such as chitosan–tripolyphosphate systems prepared by ionic gelation, have demonstrated particle sizes of approximately 200 nm, with positive surface charge favoring electrostatic stability and moderate to high entrapment efficiency.^66^ Importantly, these systems provided sustained drug release for up to 24 h after an initial burst effect, supporting the potential for localized lung therapy with reduced dosing frequency.^66,100,101^

Similarly, lipid-based nanoparticles have shown a favorable performance. In particular, SLNs have been proposed as alternatives to polymeric systems due to their faster *in vivo* degradation, which may mitigate concerns related to long-term polymer accumulation in lung tissue.^25,29,102^ Rahmanian et al. developed MTK-loaded SLNs via the hot homogenization method to enhance systemic bioavailability. DPI formulations were subsequently prepared by SD, incorporating a mannitol and lactose mixture or no carrier. SLNs presented a particle size below 100 nm and an encapsulation efficiency exceeding 99%. *In vitro* aerosol performance assessed using an NGI at 60 L/min showed that DPIs containing mannitol and lactose achieved an emitted dose greater than 93%, compared to 78% for the carrier-free formulation.^99^

Second-generation lipid systems, particularly NLCs, composed of solid and liquid lipids forming an unstructured lipid matrix, have been explored to improve drug loading capacity and physicochemical stability compared with SLNs.^103,104^ Patil-Gadhe et al. developed MTK-loaded nanostructured lipid carriers (MNLCs) using Precirol ATO 5 (solid lipid) and Capryol 90 (liquid lipid) in a 7:3 ratio, prepared via the melt-emulsification-ultrasonication method. The resulting nanoparticles exhibited an appropriate mean particle size with a homogeneous distribution. *In vitro* cytotoxicity assays confirmed their nontoxicity and compatibility with a lung A549 cell line. Nevertheless, the nanoscale dimensions posed potential limitations, including a tendency for particle aggregation and suboptimal flow properties. To address these issues, the authors implemented a dual-scale nanocomposite approach.^67^ Ullah et al. used a similar strategy to prepare MTK-loaded polymeric nanoparticles for inhaled delivery, followed by physical mixture with lactose microspheres. The study explored four different formulations containing chitosan, Tween 80, hyaluronic acid, or l-leucine, each with distinct characteristics that influenced their performance. Particle sizes ranged from 220 to 383 nm, with polydispersity indices below 0.50. Drug association efficiency varied from 46% to 86%, depending on the specific excipients incorporated. When delivered through a DPI, the formulations achieved MMAD between 1 and 2 μm and dispersed fractions ranging from 75% to 90%, indicating efficient powder dispersion from the device. Among all tested formulations, the leucine-modified nanoparticles demonstrated the most favorable performance, achieving the highest dispersed fraction of 89% from the DPI and FPF of 83%.^100^ The same authors subsequently proposed to prepare hyaluronic acid-coated nanoembedded microparticles via the freeze-drying method using mannitol or lactose as a carrier to improve the therapeutic performance of the MTK DPI product. Although both carrier-based microparticles exhibited comparable physicochemical characteristics, the lactose-embedded nanoparticles demonstrated a superior inhalation profile, with higher FPF, dispersibility, and respirable fraction.^101^

## 4 Quality Target Product Profile of Inhaled Delivery of MTK

The quality target product profile (QTPP) is an essential component of the quality-by-design (QbD) approach. The QTPP points out the quality characteristics for a drug product to ensure quality, considering drug product safety and efficacy.^105^ The next sections will address aspects related to the QTPP of DPIs, presenting studies involving MTK. Ideally, the QTPP for MTK as an inhaled local asthma therapy should evidence efficient pulmonary deposition and retention and therapeutically aligned release kinetics and stability while avoiding systemic exposure. Figure 4 summarizes the QTPP for MTK as an inhaled local asthma therapy.

**Figure 4:**
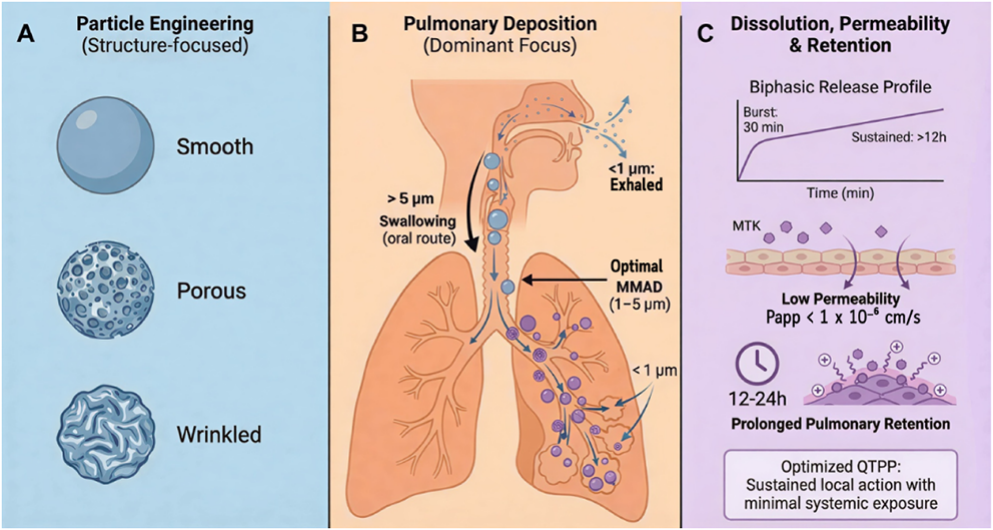
Important factors in the design of inhaled montelukast (MTK) formulations, including particle engineering, pulmonary deposition, and dissolution, permeability, and retention after deposition. (A) Representative particle morphologies are shown as smooth, porous, and wrinkled, highlighting structural features that may affect aerosol performance and lung deposition. (B) Particle size is a major determinant of deposition in the respiratory tract. Particles with aerodynamic diameters >5 μm are more likely to deposit in the upper airways and be cleared by swallowing, whereas particles <1 μm are more likely to be exhaled. By contrast, a mass median aerodynamic diameter (MMAD) in the range of 1–5 μm is generally considered optimal for deposition in the lower airways and lung periphery. (C) After deposition, formulation performance depends on the release profile, epithelial permeability, and residence time in the lung. The scheme shows a biphasic release profile, with an initial burst within 30 min followed by sustained release for >12 h, together with low permeability of MTK (P_app_ < 1 × 10^–6^ cm/s) and prolonged pulmonary retention over 12–24 h. These features are consistent with an optimized quality target product profile (QTPP) aimed at sustained local action with minimal systemic exposure. This image was created with Google AI Studio using the Nano Banana Pro model.

### 4.1 MTK Efficient Pulmonary Deposition for Asthma Treatment

Once inhaled, the site of drug deposition is fundamental for efficacy and effective treatment.^106^ Asthma has long been viewed as a condition primarily affecting the large airways, in which smooth muscle thickening leads to preferential deposition of inhaled drugs in this region.^35,107^ The distal airway region can also be affected by asthma, where luminal occlusion can occur due to multiple factors, such as excessive mucus production, smooth muscle hypertrophy, and cellular infiltrates.^108,109^ This situation worsens the disease^110^ and is also seen in nocturnal, exercise-induced, and allergic asthma.^108,111,112^ Given that asthma involves pathological processes affecting both central and peripheral lung regions, effective therapeutic treatment with MTK would ideally require drug deposition at these sites. Conveniently, the molecular target of MTK, the leukotriene receptor, is distributed throughout the lung.^13^

To ensure efficient MTK lung deposition, studies employed particle engineering strategies. In this context, the SD technique, together with surface-modifying agents such as l-leucine and PEI, is frequently employed (Tables 1 and 2). Often, adding l-leucine^48^ and PEI^17,59,60^ results in particles with wrinkled and porous surfaces, respectively. These surface features decrease the particle density, interparticle contact points, and agglomeration, thereby enhancing powder aerosolization.^113^ The nature of the SD process often yields spheroidal particles. Particle sphericity tends to result in lower powder aggregation and enhanced flowability, aerosolization, and deposition behaviors.^114^ The SD enables modulation of particle characteristics to more precisely control shape, size, morphology, and density, which are critical parameters for effective powder aerosolization and lung deposition since they influence the aerodynamic properties MMAD and FPF.

According to Table 1, the MTK DPI formulations developed consisted of microparticles with geometric diameters (D_g_) ranging from 0.9 to 12 μm, primarily spherical, with smooth, porous, or wrinkled surfaces. The reported MMAD was <5 μm, but some studies omit this crucial information. Typically, the optimal D_g_ and MMAD values for lung deposition are 1–5 μm. Although the D_g_ of some MTK DPI deviates from this specification, the resulting low-density powder (<0.4 g/cm^3^) guarantees a proper MMAD (Table 1). For instance, Patel et al. found that large, porous MTK-PLGA particles (D_g_ = 7.72 μm) had an extensive distribution throughout the lung and an MMAD of 2.51 μm, attributable to their low density resulting from internal void spaces created by PEI. In addition, these larger particles exhibited enhanced lung retention due to reduced macrophage-mediated clearance, which more effectively occurs with 1.5–3 μm particles.^17^ Using the same strategy, an advanced study of the same author produced larger porous MTK-PLGA-PEG particles (D_g_ = 10 μm) coated with heparin. These particles had an MMAD (4.14 μm) suitable for pulmonary deposition and showed improved evasion of pulmonary clearance, simultaneously ascribed to their large geometric size and PEG.^60^ Furthermore, efficient pulmonary drug deposition requires a combination of low MMAD and high FPF. Considering the MTK DPI formulations presented in Table 1, FPF was around 50%, with the highest values associated with low-density powders.^56,60^ Currently marketed available DPIs typically achieve FPF ranging from 10% to 50% of the labeled dose.^41,115^

Table 2 shows five MTK DPI formulations based on nanostructured particles for pulmonary drug delivery. To overcome issues inherent to particles at the nanoscale, such as aggregation, exhalation, and cellular clearance, the MTK DPI nanoparticles were admixed with larger carriers.^67,100,101^ The exception was the study from Inamdar et al., which developed an isolated MTK nanoparticle.^66^ In this case, the absence of powder aerodynamic characterization precludes assessing its suitability for use as a DPI formulation. Conversely, the studies by Patil-Gadhe et al. and Ullah et al. provided a detailed characterization of the formulation’s physicochemical and aerodynamic properties.^67,100,101^ Although these studies employed distinct nanoparticle matrices (polymeric and lipidic) and methods for producing the MTK DPI nanostructured formulation, they nevertheless relied on sugar carriers, specifically mannitol and lactose. Aerodynamically, the MTK nanoformulation developed by Patil-Gadhe et al. exhibited an appropriate MMAD and high FPF (∼70–90%) across two flow rates (30 and 60 L/min). Although these results should be interpreted cautiously in clinical practice, they are potentially promising for patient treatment with varying degrees of respiratory impairment, such as asthmatics. Additionally, the *in vitro* deposition profile indicated preferential particle deposition in stages 3–6, which *in vivo* would correspond to the tracheobronchial and distal bronchial regions.^67^ Using the same approach, Ullah et al. prepared several MTK nanoformulations with lactose as a carrier and ultimately identified the formulation containing 10% l-leucine as the most potent. This formulation achieved a higher MTK delivered dose and FPF (83%) than the others, owing to the hydrophobic and glidant properties of l-leucine, which decreased interparticle cohesion and aggregation during aerosolization.^100^

### 4.2 Local Acting MTK Formulations Should Be Retained in the Lungs

Achieving adequate aerodynamic performance alone does not guarantee optimal therapeutic outcomes, because postdeposition events, such as interactions with mucus and cellular absorption, are crucial in determining how long the drug is retained in the lungs.^116,117^ A poor particle-mucus interaction and efficient absorptive process abbreviate drug clearance from the pulmonary environment. Considering that MTK DPIs are designed for local action, an increased drug residence time will benefit treatment.

Mucins are negatively charged glycoproteins of the mucus, in which positively charged particles or ions bind electrostatically to them.^116^ Studies explore this phenomenon to prolong the drug residence time via adhesion to and entrapment within the mucus layer. In the context of MTK intended for DPI, Panchal et al. demonstrated that increasing the proportion of the cationic polymer chitosan in the MTK microspheres exacerbated mucoadhesion.^55^ This same polymer was employed by Ullah et al., and positively charged MTK was produced, but the authors did not perform any mucoadhesion evaluation.^100,101^

Mucoadhesion can also occur via hydrogen bonding, hydrophobic interactions, and van der Waals forces, depending on the particle surface chemistry.^118,119^ Mahajan and Gundare justified that the mucoadhesion and extended pulmonary retention (6 h) of the negatively charged MTK microparticles result from the interaction between the xyloglucan polymer and mucin. The structural similarity between xyloglucan and mucin facilitates physical particle penetration into the mucus network, enhancing the affinity and retention.^64,120^

### 4.3 MTK Dissolution

For inhaled drugs to exert their therapeutic effect, they must first dissolve in the lung lining fluid, enabling subsequent diffusion, cellular uptake, absorption, and interaction with target receptors. Dissolution performance depends on the drug’s physicochemical properties as well as formulation factors, including composition, particle size, morphology, and solid-state characteristics, all of which are influenced by the manufacturing process.^121^

MTK is a highly lipophilic compound (log *P* 8.79) with a 586.18 g/mol molecular weight.^122^ The drug exhibits pH-dependent solubility due to its two ionizable groups. Although the composition of lung lining fluid varies along the respiratory tract, its pH in healthy individuals remains constant at around 6.7.^21^ At this pH, MTK would be partially ionized, which slightly enhances its aqueous solubility.^123^ The MTK free acid is poorly water-soluble, ranging from 0.2 to 0.5 μg/mL at 25 °C.^123^ Based on its low solubility and high oral permeability (5 × 10^–6^ cm/s), MTK is Class II in the Biopharmaceutical Classification System (BCS).^47,124^ The development of a BCS suitable to the pulmonary route has been widely addressed in the literature; however, it has not yet gained regulatory status.^19,125,126^ Commonly, poorly soluble substances, such as MTK, are available as salt forms. MTK salt sodium has a marked increase in water solubility, reaching 100–1000 μg/mL,^27^ and is freely soluble in alcohol and methylene chloride.^127^ Its amorphous nature favors solubility.^128^

Various formulation strategies have been proposed to improve the MTK dissolution. Fast-release SD inhaled microparticles formulated with hydrophilic excipients such as mannitol, ovalbumin, and lecithin consistently exhibit rapid and nearly complete dissolution within minutes in simulated lung fluids.^48,56^ Notably, even spray-dried drugs without excipients also showed markedly enhanced dissolution due to particle engineering, with 95% release within the first 5 min.^56^ Although improving drug dissolution for pulmonary delivery can ensure rapid local availability and reduce macrophage uptake of undissolved particles, excessively rapid dissolution may also lead to rapid systemic absorption. This, in turn, lowers the lung-to-plasma concentration ratio and reduces pulmonary retention, which is undesirable for treating local respiratory conditions, such as asthma.^129^

Locally acting formulations often possess properties, such as low solubility or slow dissolution rate, limited permeability, or strong tissue retention, that potentially restrict their absorption into the systemic circulation and favor pulmonary residence.^117,130^ Several controlled-release strategies have been proposed to prolong MTK lung residence time. In fact, most formulations reported in the literature exhibit a characteristic biphasic release pattern consisting of an initial burst effect followed by sustained release. This burst effect, typically occurring within the first 30 min, was attributed to the superficial presence of MTK adsorbed on the particle surface, which is rapidly exposed to the dissolution medium due to the high surface area-to-volume ratio of the particles.^59,66,100^ The sustained phase, however, reflects the diffusion-controlled release from polymeric or lipid matrices.

Both polymer selection and concentration in the formulation significantly influence the extent of the sustained release. Polymeric systems based on natural polysaccharides, such as chitosan and xyloglucan, typically provided sustained drug release over hours.^55^ Co-spray drying of xyloglucan with lactose resulted in a sustained MTK release of approximately 90% over 167 min.^64^ In contrast, MTK DPI systems with synthetic polymers generally enable longer release kinetics. Dual-drug particulates composed of MTK and low-molecular-weight heparin, prepared using PLA, PLGA, and PEG, displayed biphasic release with sustained drug release extending up to 4 days.^60^ This behavior agreed with previously reported release profiles of drugs formulated in similar polymeric matrices.^59^ The underlying mechanism involved surface pores that facilitated the initial burst release, while the core drug reservoir provided sustained release.^17,59^ Lipid-based systems, particularly nanostructured lipid carriers formulated as DPI powders, provided pronounced retardation of MTK release. MNLC-DPI formulations released less than 20% of the drug within the first 2 h and required over 24 h for complete dissolution, in contrast to the rapid release of aqueous drug solutions.^67^

In terms of polymer content, higher amounts are associated with slower drug release rates. Sustained-release MTK microparticles prepared with Eudragit RS100 demonstrated an inverse correlation between polymer concentration and release rate, with not less than 79% released at 12 h.^61^ Ullah et al. confirmed this behavior with chitosan particles and observed that coating with hyaluronic acid and l-leucine provided more controlled release, following the Higuchi model, indicating a diffusion-controlled mechanism consistent with Fick’s law.^100^

The literature shows a clear link between the formulation strategy and pharmacokinetic outcomes. Fast-release systems enhance early systemic exposure and are preferable for a rapid onset of action, while polymeric and lipid-based sustained-release particles, despite an initial burst, favor prolonged pulmonary residence. Thus, for DPI systems aimed at chronic asthma management, such as MTK, sustained-release strategies better support maximal lung targeting and minimal systemic exposure.

### 4.4 Permeability and Absorption

Absorptive clearance occurs by permeation of the drug from the epithelial lining fluid in the lumen into and across the lung epithelium,^126^ and it is dependent on the substance’s molecular properties, such as permeability, lipid partitioning, and affinity to active and passive transporters.^121^ Aerosol drugs are absorbed through the pulmonary epithelium primarily by diffusion and possibly via transporters. Hydrophilic drugs (log *P* < 0) are absorbed via the paracellular route, typically within approximately 1 h. In contrast, lipophilic drugs (with log *P* > 0) are rapidly absorbed via the transcellular route.^19^

Permeability is a critical attribute for inhaled drugs, as it influences drug clearance from the airway lumen rather than solely determining systemic absorption. In this context, high tissue affinity serves as an additional mechanism for pulmonary retention, which may enhance the efficacy of locally acting drugs.^126^ The physicochemical properties of MTK disfavor absorption by passive diffusion: its high molecular weight and strong lipophilicity suggest that, once partitioned into the cell membrane, its release would be hindered.^16^ Muraki et al. observed that MTK inhibited both the bronchoconstriction and arterial constriction induced by intravenous leukotrienes, suggesting that inhaled MTK exerts its effect by penetrating the submucosa of the airways.^45^ Wang et al. proposed an HPLC-MS/MS method for the quantification of MTK in an *in vitro* cell-based pulmonary model (Calu-3 cells) to determine the drug’s permeability.^131^ The results indicated that MTK has a low permeability in the Calu-3 monolayer, since the coefficient of apparent permeability (P_app_) was <1 × 10^–6^ cm/s.^125,130^ All *in vivo* studies of inhaled MTK pharmacokinetics identified in this review used rat models, which may limit extrapolation to the human pulmonary context (Table 3).

**Table 3:** *In Vivo* Pharmacokinetic Performance of Montelukast (MTK) Formulations in Animal Models^a^

ref	formulation	dose (mg/kg)	model	via	*C*_max_	*T*_max_ (h)	*t*_1/2_ (h)	AUC_0–*t*_	*t* (h)	AUC_0–∞_
 ^17^	Pure drug	2.1	Rats	IT	1.89 ± 0.54 μg/mL	NR	6.22 ± 1.62	1.47 h·μg/mL	12	NR
	Pure drug suspended in PBS	2.1	Rats	oral	0.46 ± 0.23 μg/mL	NR	3.46 ± 1.23	0.45 h·μg/mL	12	NR
	Pure drug	2.1	Rats	IV	5.21 ± 0.81 μg/mL	–	7.41 ± 1.78	1.83 h·μg/mL	12	NR
^64^	Microspheres	1	Rats	IT	406.21 ± 35.60 ng/mL	1	NR	103.38 min·ng/mL	6	234.99 min·ng/mL
	Pure drug	1	Rats	oral	347.51 ng/mL	0.25	NR	22.04 min·ng/mL	6	23.44 min·ng/mL
^67^	MNLC-DPI	0.05	Rats	IT	2047.09 ± 112.35 ng/mL	8	21.49	4778.28 ± 335.24 h·ng/mL	24	29478.36 h·ng/mL
	Pure drug solution	0.05	Rats	IT	1750.00 ± 175.00 ng/mL	2	6.40	446.00 ± 22.30 h·ng/mL	24	2506.22 h·ng/mL
^101^	Mannitol-embedded nanoparticles	3	Rats	IT	46.57 ± 9.09 μg/mL	19.83 ± 1.48	12.96 ± 0.33	856.43 ± 15.01 h·μg/mL	24	NR
	Lactose-embedded nanoparticles	3	Rats	IT	46.20 ± 6.03 μg/mL	20.96 ± 2.08	13.71 ± 0.25	835.23 ± 13.01 h·μg/mL	24	NR
	Pure drug solution	3	Rats	IT	80.91 ± 5.08 μg/mL	9.25 ± 0.19	6.20 ± 0.13	1488.92 ± 10.01 h·μg/mL	24	NR

a NR = not reported, IT = intratracheal, IV = intravenous, PBS = phosphate-buffered saline, and MNLC = montelukast nanostructured lipid carrier.

The studies demonstrated that the route of administration plays a critical role in pulmonary drug accumulation and systemic exposure. Direct pulmonary delivery of MTK, regardless of the inhalation system used, led to a marked improvement in the pharmacokinetic profile compared with oral administration. Patel et al. evaluated the absorption of free MTK in male rats following oral, pulmonary, and intravenous administration (Table 3) and showed that inhaled MTK provides enhanced pulmonary exposure compared with oral dosing. For instance, an intratracheally aerosolized MTK solution achieved an absolute bioavailability exceeding 80%, representing an improvement over the approximately 25% bioavailability observed with oral dosing in rats. The pharmacokinetic parameters further supported the metabolic stability advantages of MTK pulmonary delivery. Inhaled MTK exhibited a higher peak plasma concentration (*C*_max_ ∼ 2 μg/mL) and prolonged elimination half-life (*t*_1/2_ ∼ 6 h) compared to oral administration (*C*_max_ ∼ 0.5 μg/mL; *t*_1/2_ ∼ 3.5 h). These findings closely resemble the pharmacokinetic profile observed with intravenous administration, indicating that inhaled delivery effectively circumvents first-pass hepatic metabolism and thereby allows for lower dosing, potentially reducing systemic side effects and enabling longer dosing intervals.^17^ Although the data suggest an efficient pulmonary absorption of the free drug following intratracheal administration, the lack of AUC_0–∞_ determination and the restriction of plasma sampling to 12 h limit a more definitive assessment of the elimination phase and its contribution to the overall systemic exposure. Accordingly, differences observed in apparent elimination half-life among administration routes should be interpreted cautiously, as they may arise from limitations in defining the terminal phase rather than from intrinsic, route-dependent changes in systemic clearance.

Considering the pharmacokinetic behavior of MTK DPI (Table 3), following intratracheal instillation of xyloglucan-based MTK microspheres, a maximum plasma concentration (*C*_max_ ∼ 410 ng/mL) slightly higher compared with orally administered pure drug (*C*_max_ ∼ 347 ng/mL) was observed. Notably, a much more pronounced increase in systemic exposure was observed, and authors attributed it to the polymeric nature and mucoadhesive properties of the microspheres.^64^ Additionally, the delayed *T*_max_ after intratracheal administration supports a slower and more sustained absorption process from the pulmonary lumen. In contrast, oral administration was characterized by a rapid attainment of peak plasma concentrations and minimal contribution from the terminal phase, consistent with limited and transient systemic exposure. The absence of elimination data impairs a comprehensive characterization of the kinetics governing the terminal phase, limiting conclusions about systemic elimination and potential dosing interval advantages.

Patil-Gadhe et al. demonstrated that encapsulation of MTK in NLCs markedly altered its pulmonary disposition compared to an aqueous solution (Table 3). Following intratracheal administration, drug levels were quantified in bronchoalveolar lavage fluid (BAL) as a surrogate of unabsorbed drug available for subsequent uptake and in lung homogenates (LH) to assess absorbed drug within lung tissue. While the aqueous formulation exhibited rapid pulmonary absorption and clearance, the MNLC-DPI maintained sustained drug availability in BAL and prolonged drug retention in LH, with detectable levels for up to 12 h postdose, consistent with modified absorption kinetics and extended lung residence. The aqueous formulation reached peak lung concentrations rapidly (*C*_max_ ∼ 1750 ng/g at 2 h), followed by rapid clearance and undetectable levels by 24 h. In contrast, the nanoencapsulated formulation achieved slightly higher peak concentrations (*C*_max_ ∼ 2050 ng/g) with a delayed *T*_max_ (8 h). This kinetic profile, together with the extended elimination half-life (21.49 h), is consistent with sustained pulmonary release and extended residence time. The authors attributed this enhanced performance to suitable aerodynamic behavior, reduced cellular clearance, and improved powder dispersibility.^67^

Ullah et al. similarly reported higher values of *T*_max_, mean residence time, and half-life for MTK-chitosan nanoparticle-embedded microparticles administered via the intratracheal route compared to the pure drug solution administered by the same route, with approximately a 2-fold increase in these parameters relative to the aqueous montelukast solution. In contrast to the aforementioned studies, however, the pure montelukast solution yielded a higher *C*_max_ (∼80 μg/mL) than the nanoparticle-embedded microparticles (∼46 μg/mL). The authors attributed this effect to drug sustained release from the chitosan nanoparticles.^101^

Although pharmacokinetic parameters are reported for comparative purposes, differences in dose, route of administration, analytical methodologies, and sampling intervals among the studies prevent direct quantitative comparisons. In this context, a consistent pattern emerges in which inhalable formulations, particularly those based on particulate or nanostructured delivery systems, promote higher and more prolonged systemic exposure compared to oral administration of the free drug, underscoring the critical role of particle engineering and pulmonary delivery in modulating MTK pharmacokinetics.

Analysis of sustained-release technologies further highlights formulation-dependent differences in absorption kinetics. Xyloglucan-based microspheres, characterized by a relatively short *in vitro* dissolution time (∼139 min), exhibited an early *T*_max_ following intratracheal administration, consistent with a limited temporal extension of drug release and absorption.^64^ In contrast, NLC formulation, which displays markedly prolonged *in vitro* dissolution profiles (∼24 h), was associated with a substantially delayed *T*_max_, indicating slower systemic input after pulmonary administration.^67^ Notably, these pharmacokinetic features of MNLC-DPI were observed despite the use of a substantially lower administered dose compared to the microsphere-based formulation, underscoring the strong influence of formulation design on the rate and extent of pulmonary absorption. While direct quantitative comparisons remain limited by differences in dose and study design, the qualitative correspondence between *in vitro* dissolution behavior and *in vivo* *T*_max_ supports a formulation-driven modulation of absorption kinetics.

Importantly, these pharmacokinetic findings are aligned with independent pharmacodynamic studies demonstrating comparable therapeutic efficacy of inhaled MTK at doses 10-fold lower than those required orally.^48^ Together, these observations suggest that the enhanced exposure achieved via the pulmonary route reflects greater absorption efficiency, which may underlie the dose-sparing effects reported for inhaled formulations.

### 4.5 Stability

MTK is highly susceptible to light and oxygen degradation.^132–136^ DPI prepared by SD represents the most extensively investigated platform for the pulmonary delivery of MTK and offers advantages from a formulation-stability perspective. Compared to liquid-based inhalation systems, DPIs are less prone to hydrolytic degradation, chemical instability, and component incompatibility,^137,138^ which are particularly critical concerns for this drug.

MTK is an amorphous compound, and SD processing maintains this solid state.^48,54,56,67^ While amorphous systems often exhibit improved dissolution behavior compared to their crystalline counterparts, they are intrinsically metastable and sensitive to moisture.^139^ Adsorbed water can act as a plasticizer, lowering the glass transition temperature and, consequently, promoting solid-state transitions and recrystallization.^140,141^ Increased particle–particle interactions due to capillary forces can also lead to enhanced powder agglomeration, reducing aerosolization,^142^ causing batch-to-batch performance variability, and significantly impacting formulation stability.^143^ Consequently, excipient selection is critical in spray-dried MTK formulations to restrict molecular mobility and to enhance physical stability. Polymers and functional excipients such as hydroxypropyl methylcellulose,^144,145^ polyvinylpyrrolidone,^144,145^ and l-leucine^89,92,146^ are frequently incorporated to enhance physical stability, powder dispersibility, and aerosol performance.

A previous study reported by Moussa et al. demonstrated that spray-dried amorphous MTK without any additives produced a powder prone to agglomeration and stickiness within 2 days of preparation, even when stored in desiccators. To address these issues, the authors investigated the incorporation of mannitol, l-leucine, and a mannitol-leucine mixture as stabilizing excipients. Three formulations were prepared and assessed for total drug content and *in vitro* pulmonary deposition after 3 and 6 months of storage in desiccators at room temperature (25 ± 2 °C). All powders remained stable over the storage period, with no significant changes in drug content or deposition performance. Physicochemical analyses by differential scanning calorimetry and X-ray powder diffraction revealed that the spray-dried powders retained crystalline characteristics, primarily due to the inclusion of l-leucine and mannitol at high concentrations. This crystallinity was considered a key factor in improving stability and enhancing powder flow properties.^56^

Abdel-Gawad et al. evaluated the six-month shelf life stability (25 ± 2 °C) of soft agglomerate microparticles with mannitol, l-leucine, and lecithin for inhalable MTK. The drug association efficiency remained consistently above 95% throughout the storage period, with preserved aerosol performance characteristics, as evidenced by maintained drug deposition patterns across the twin-stage impinger stages. The authors attributed the observed stability to the crystalline nature of the SD powder, which prevented the aggregation and particle fusion commonly associated with amorphous SD formulations, and to the low moisture content achieved (0.04%) through the SD process, which minimized hydrolytic degradation pathways.^48^

## 5 Clinical Trials and Patents

A total of 178 clinical studies were identified, with the majority (*n* = 153) classified as completed and investigating the use of MTK mainly for the treatment of asthma, allergic rhinitis, and other related respiratory conditions. These studies generally involved adult and pediatric populations, including specific subgroups such as smoking asthmatic patients, and evaluated MTK either as monotherapy or in combination with other drugs, most often in association with ICSs. Only three (*n* = 3) of the identified clinical trials evaluated inhaled MTK, two of which are associated with the studies published by Philip et al.^23,24^

The first was a phase I study, initiated in August 2007, that assessed the safety, tolerability, and pharmacokinetics of a DPI formulation in participants with mild to moderate asthma. Multiple dose levels (0.1, 0.3, 1, 3, and 10 mg) were investigated and compared with placebo (NCT00636207).^147^ The second study, initiated in May 2008, compared inhaled Mometasone, an ICS, combined with inhaled MTK (DPI, 1 mg) versus ICS therapy alone in patients with chronic asthma (NCT00666679).^148^ This study was also published by Philip et al. and demonstrated that the combination of MTK with ICS was more effective than placebo plus inhaled Mometasone.^24^ The third study, initiated in July 2008, was a phase I placebo-controlled clinical trial that demonstrated rapid and sustained bronchodilation in patients with chronic asthma following a single administration of inhaled MTK (25–1000 μg/dose). The onset of action was approximately 2 h for the lowest effective dose (100 μg) and 20 min for the highest dose (1000 μg), with effects persisting for up to 24 h at both doses (NCT00739297).^149^ This study was also published by Philip et al., reporting that the tolerability of inhaled MTK was comparable to that of placebo.^23^ All studies have been completed.

Despite the inclusion of these three trials, most of the clinical research on MTK continues to rely on oral administration. No large-scale efficacy trials of pulmonary MTK formulations (e.g., inhalable powders, aerosols) were identified beyond early phase and combination designs.

With respect to patents, 859 were identified, all of which were filed in the USA, distributed by years as follows: 2020 (*n* = 158), 2021 (*n* = 144), 2022 (*n* = 151), 2023 (*n* = 142), 2024 (*n* = 143), and 2025 (*n* = 121). The main applicants were Bristol Myers Squibb (*n* = 41), Incyte Corp. (*n* = 29), Kymera Therapeutics Inc. (*n* = 28), Brigham and Women’s Hospital Inc. (*n* = 22), Massachusetts Institute of Technology (*n* = 19), GlaxoSmithKline IP Development Ltd. (*n* = 18), and Pfizer (*n* = 17). When the results were filtered by patent families, that is, groups of patent documents originating from the same priority application, the total number of patents was reduced to 578. Using an artificial intelligence setting (ChatGPT Plus 5.2), the total was filtered. Finally, only three (*n* = 3) patents fit the criteria, and after careful reading of those patents, none of them was in agreement with the use of MTK powder for inhalable devices. Caliendo et al. (US 12338266 B2) developed esters of MTK with corticosteroids and mentioned the possibility of delivering this drug through DPI.^150^ Chandler (US 11103500 B2) proposed the use of levocetirizine and MTK for treating lung and brain injury and proposed that the use of this formulation may be for inhalation.^151^ Lastly, Caliendo et al. (US 12453727 B2) studied salts of MTK with β2 adrenergic agonists for the treatment of inflammatory pathologies, obstructive pathologies, and allergen-induced (which asthma can be included in) and proposed inhalation for it.^152^ These findings highlight a clear research gap in MTK-based asthma management via pulmonary delivery, since most patents only mention inhalable administration as a possibility and do not provide detailed development or optimization of inhalation devices. Even when patent applications provide limited or no detailed information, the pursuit of broad patent protection is a recurring strategy among applicants seeking to preserve exclusivity in the pharmaceutical market. This situation led to the inclusion of these patents in the search criteria, but they fall outside the scope of the present review. The scarcity of advanced clinical trials and the absence of specific inhalable MTK technologies suggest that, despite promising pharmacological rationale, pulmonary delivery of MTK remains at an early translational stage.

## 6 Comparative Summary and Practical Insights

The development of an inhalable MTK formulation for asthma therapy is primarily oriented toward delivery via DPIs, which require powders with carefully engineered physicochemical and aerodynamic properties. Particle engineering strategies are central to achieving formulations composed of low-density, spherical particles with an MMAD within the optimal range of 1–5 μm and high FPF, thereby ensuring efficient pulmonary deposition. SD-based microparticulate systems are particularly advantageous in this context, as they rely on well-established, scalable manufacturing processes and benefit from a consolidated regulatory framework, which facilitates their translation into commercial products. Although nanostructured delivery systems have demonstrated promising performance, concerns related to long-term safety, regulatory acceptance, and large-scale manufacturability may limit their near-term application in inhaled MTK therapy.

Drug loading and its proportion within the formulation exert a marked influence on powder performance, highlighting the importance of particle engineering and excipient selection to address intrinsic limitations of MTKs, such as hygroscopicity, physical instability, and their amorphous character. The incorporation of multifunctional excipients contributes to reduced particle density, improved powder dispersibility, and enhanced physical stability, including the promotion of favorable solid-state characteristics.

From a biopharmaceutical perspective, inhalable MTK formulations are expected to exhibit a sustained dissolution profile, often characterized by an initial burst dissolution, followed by prolonged drug release. This behavior is well-aligned with the clinical use of MTK as a maintenance therapy for asthma, rather than for acute symptom relief. In addition, the lipophilic nature and limited permeability of MTK support its retention within the pulmonary epithelium, which is consistent with a localized mechanism of action in the lungs. *In vivo* studies have shown, in selected preclinical models, that inhaled microparticulate systems can enable pulmonary delivery of MTK and result in pharmacokinetic profiles distinct from those observed following oral administration, although direct quantitative comparisons across studies are limited by methodological heterogeneity. Overall, particle engineering approaches that allow controlled drug dissolution and prolonged pulmonary residence are expected, on the basis of these preclinical observations, to support a pharmacokinetic profile potentially favorable for chronic asthma management; confirmation of dose reduction and of improved dosing regimens in patients, however, remains to be demonstrated in adequately powered clinical studies.

Among the inhalable MTK formulations reviewed, those achieving FPF above 50% with MMAD in the 1–3 μm range share a common design principle: the deliberate reduction of apparent particle density through porosity or surface roughness, rather than size reduction alone. Large porous PLGA/PLA microparticles incorporating PEI and leucine-modified polymeric microparticles illustrate this principle, with reported tap densities below 0.4 g/cm^3^ and high emitted doses. In contrast, formulations that did not include systematic aerodynamic characterization or that reported FPF below 30% should be regarded with caution, as they cannot yet be considered suitable for DPI development without further evidence.

MTK is administered orally at 10 mg/day, which has often been used as a conservative reference dose when designing inhaled formulations and assessing their translational feasibility. However, direct equivalence between oral and pulmonary dosing cannot be assumed, since pulmonary delivery may substantially alter local drug exposure, systemic absorption, and first-pass metabolism. Although clinical practice demonstrates that relatively high powder loads can be technically administered through dose fractionation and multiple inhalation acts, the clinically effective inhaled MTK dose in humans remains unknown. In the broader pulmonary drug delivery literature, dose reductions relative to oral administration have been proposed for some compounds due to improved local delivery and reduced first-pass metabolism, in some cases reaching approximately 10–20% of the corresponding oral dose.^22,114^ Under this rationale, studies suggest that pulmonary administration of MTK may potentially achieve therapeutic effects at lower doses than those used orally. However, no standardized dose conversion between oral and inhaled administration currently exists, and the magnitude of potential dose reduction remains highly drug-dependent. Therefore, dose feasibility should not be interpreted solely on the basis of oral-equivalent calculations but rather in the context of future pharmacokinetic, pharmacodynamic, and clinical validation studies.

Although clinical practice shows that high doses are feasible for inhaled products, the required inhaled dose is expected to be reduced to about 10–20% of the oral dose (i.e., approximately 1–2 mg of MTK per day), as reported.^22,114^ It must be emphasized that such dose-sparing has not yet been demonstrated in humans for MTK; the few preclinical observations available in the studies reviewed here are encouraging but do not allow firm extrapolation. From a dose perspective, many studies presented may be considered acceptable for the development of a commercial MTK DPI. However, the dose feasibility of inhaled MTK should not be regarded as an intrinsic property of a given formulation but rather as contingent on the clinically validated dose. Accordingly, future studies in this field would benefit from reporting both the absolute drug loading and the corresponding powder mass required to achieve target doses. Despite the well-known amorphous and hygroscopic nature of MTK and the additional stability concerns introduced by polymeric or lipid-based carriers, only two^48,56^ of the 20 reviewed studies reported stability assessments over three- and six-month periods, although only in a preliminary manner and without following the requirements or rigor of an official compendial guideline. The current evidence base on stability is therefore a major translational gap rather than a solved problem.

SD is the most frequent and most translationally mature production method in the reviewed data set, supported by an established Good Manufacturing Practice framework for inhaled products. More elaborate processes, such as spray-freeze-drying, double emulsion followed by lyophilization, or sequential ionic gelation followed by SD for nanoin-microparticulate systems, yield attractive *in vitro* performance but introduce additional scale-up, validation, and impurity-control burdens that have not yet been demonstrated for MTK. Carrier polymers such as PLGA, although extensively explored in this review, currently lack precedent in commercially approved inhaled products, which represents a regulatory consideration that should be made explicit when discussing translational potential.

As already acknowledged in the pharmacokinetic section of this review, only four of the reviewed studies reported *in vivo* pharmacokinetic data for inhaled MTK, and direct quantitative comparisons across them are precluded by heterogeneity in doses, routes, sampling schemes, and analytical methods. Pharmacodynamic evidence is even more restricted, with only a small number of studies addressing functional outcomes. Therefore, claims of superior pharmacokinetic profiles or sustained local activity should be presented as expectations supported by individual preclinical observations rather than as established translational outcomes.

The cumulative picture emerging from this review helps explain why no inhaled MTK product is currently commercialized despite more than a decade of formulation research. The three patents retrieved that mention inhalation as a possible route do not actually disclose developed inhaled MTK formulations or device strategies, and only three completed early-phase clinical studies have evaluated inhaled MTK to date.

## 7 Conclusion and Future Perspectives

Pulmonary delivery of MTK demonstrates promising potential as an alternative to conventional oral administration, offering the potential to achieve targeted drug deposition in the lungs, bypassing first-pass metabolism, and reducing systemic side effects. Advances in formulation science and particle engineering have enabled the design of both micro- and nanostructured systems capable of enhancing aerodynamic performance, drug stability, and local bioavailability.

Several strategies have been developed to incorporate MTK into micro- and nanoparticulate systems for the treatment of asthma. Most studies employed DPIs based on microparticulate formulations. SD was the most commonly used technique, and several formulation strategies, such as the production of porous particles, were explored to improve the aerodynamic behavior and to reduce macrophage uptake. The combination of surface modification and mucoadhesive polymers, including chitosan and xyloglucan, has further contributed to a prolonged residence time and improved drug retention at the site of action. In parallel, nanocarrier systems, including polymeric nanoparticles, SLN, and NLC, have demonstrated the potential to improve pulmonary bioavailability by providing sustained drug release and extended residence time within the lungs.

Despite these results, significant challenges remain to be addressed before clinical translation can be achieved. Stability concerns, especially during storage and aerosolization, along with scalability and reproducibility of production methods, continue to represent critical barriers. Although three completed clinical studies evaluating MTK-based inhaled therapies have been identified, the overall availability of robust *in vivo* and clinical data is still limited, which may delay regulatory approval and commercialization.

Future research could also focus on combination inhaled therapies or codelivery systems capable of targeting multiple inflammatory pathways involved in the disease. Such strategies may improve therapeutic efficacy and patient adherence while expanding the clinical utility of inhaled MTK.

Finally, it is important to distinguish between outcomes that have been demonstrated in the studies reviewed here and those that remain expected on the basis of general pulmonary delivery principles. Particle engineering strategies have reproducibly achieved aerodynamic profiles compatible with deep lung deposition for MTK, and selected formulations have demonstrated, in preclinical models, pharmacokinetic profiles distinct from those obtained after oral administration. However, evidence of clinically superior efficacy, reduced systemic adverse events in patients, dose-sparing in humans, or sustained once-daily local activity in chronic asthma is currently limited to early-phase or preclinical data. The translation of these formulation advances into approved inhaled MTK products will therefore require not only further clinical validation but also explicit attention to the regulatory, toxicological, and manufacturing barriers identified throughout this review.

## 8 Methodology

This work is a structured narrative review of the literature on the formulation and technological strategies for the pulmonary delivery of montelukast (MTK), complemented by a search of clinical trial registries and patent databases.

### 8.1 Literature Search on Formulation Studies

The search was conducted up to August 09, 2025, in three electronic databases: PubMed, Scopus, and Web of Science (WoS, Clarivate Analytics), without restrictions on year. In addition, a manual search of the reference lists of relevant articles was conducted on Google Scholar. Only articles published in English were included. The search used the following MESH terms and the respective Boolean terms: (montelukast AND (“drug delivery system” OR “drug formulation” OR carrier*) AND (inhalation OR pulmonary OR “dry powder inhaler” OR DPI OR aerosol)), adapted to each database’s specificity.

Studies were included if they addressed the development or evaluation of MTK formulations intended for pulmonary delivery, including dry powder inhalers, nebulized systems, pressurized metered-dose inhalers, or aerosol-based approaches. Eligible studies comprised experimental investigations (*in vitro*, *in vivo*, or clinical) focusing on drug delivery systems, particle engineering, formulation strategies, or inhalation device performance and were related to asthma treatment outcomes or mentioned this disease as a target.

Studies were excluded if they investigated MTK administered via nonpulmonary routes (e.g., oral or intravenous), lacked a formulation or drug delivery component, or focused solely on pharmacological or clinical aspects without addressing technological development. Review articles were excluded from the primary analysis but were considered for contextual discussion. Initially, article titles and abstracts were read to verify their fit with the previously mentioned criteria, and if necessary, the full articles were read.

A total of 143 records were initially identified across the four sources (PubMed, 13; Scopus, 115; Web of Science, 11; Google Scholar, 4). After removal of duplicates (*n* = 19), 124 records remained for title and abstract screening, of which 102 were excluded for not meeting the eligibility criteria. The remaining 22 records were assessed for full-text eligibility, and 02 were excluded due to different therapeutic indications. This procedure resulted in a final inclusion of 20 studies addressing MTK formulations for pulmonary delivery, which formed the basis of the analysis presented in this review.

### 8.2 Clinical Trial Search

A search for clinical studies involving MTK was conducted in the ClinicalTrials.gov and ClinicalTrialsRegister.eu databases. The analysis included exclusively registered interventional studies, regardless of study status (ongoing, completed, or discontinued), with a focus on therapeutic indications, type of intervention, and route of administration of MTK. The search strategy included the combination of the MeSH terms “montelukast” AND “asthma” AND “inhalation”. The search period covered records from database inception until November 14, 2025, the date on which the search was conducted.

### 8.3 Patent Search and AI-Assisted Filtering

A patent search was also conducted on Lens.org to identify patents related to MTK for inhalation. The analysis focused on the two main filing jurisdictions, the United States of America (USA) and China. In addition, the search strategy included the combination of the MeSH terms “Montelukast” and “Inhalation”, and AI setting (ChatGPT Plus 5.2) was used to help filter the results.

The following prompt was used: “Patent analysis should be conducted using an approach in which the model operates as an expert in technological prospecting within the pharmaceutical field. A spreadsheet containing previously retrieved patent documents from keyword searches will be provided, including fields such as title and abstract. The objective of the analysis is to rigorously identify which patents are directly related to the use of MTK via inhalation. To this end, predefined eligibility criteria must be applied. Only patents that simultaneously meet the following two requirements will be considered relevant: (i) explicit mention of the drug MTK and (ii) a clear description of its administration via inhalation or pulmonary delivery. The identification of the route of administration should consider technical terms associated with respiratory delivery, including, but not limited to, inhalation, inhaled, pulmonary delivery, respiratory administration, lung delivery, aerosol, dry powder, DPI, MDI, and nebulization. Patents that only mentioned MTK in the context of oral or systemic use were excluded. In addition, documents that included inhalation-related terms but did not clearly link them to the drug’s administration were not considered relevant. The analysis should rely on the title and abstract fields, focusing on the context in which the terms are used rather than their isolated occurrences. Patents should be classified in a binary manner as ‘Relevant’ or ‘Not relevant,’ with the option to include an intermediate category (‘Unsure’) in ambiguous cases. As an output, a new spreadsheet should be generated containing the selected patents, with an additional column for classification and another for justification. The analysis should be conducted conservatively, prioritizing specificity and minimizing false positives, thereby characterizing this step as an initial screening based on title and abstract”.

Initially, an Excel spreadsheet with no family distinction was created, followed by a family-specific patent spreadsheet. For AI analysis, this last one was used. On the spreadsheet, the following data were available: Jurisdiction, Kind, Display Key, Lens ID, Publication Date, Publication Year, Application Number, Application Date, Title, and Abstract. The AI tool evaluated the patents only based on their titles and abstracts. In sequence, human analyses were conducted to determine whether the AI screening met the criteria for using MTK via inhalation for asthma treatment. These analyses focused on the patent’s title, abstract, and claims. The search period was limited from January 1, 2020, to December 31, 2025, and only granted and active patents were considered.

## Abbreviations

ACI: Andersen Cascade Impactor
AI: artificial intelligence
API: active pharmaceutical ingredient
AUC: area under the curve
BAL: bronchoalveolar lavage
CD: cyclodextrin
CysLT: cysteine leukotriene receptors
DPI: dry powder inhaler
ED: emitted dose
FDA: Food and Drug Administration
GSD: geometric standard deviation
FPF: fine particle fraction
ICS: inhaled corticosteroids
IL: interleukin
HBCD: highly branched cyclodextrin
HFA: hydrofluoroalkane
HPLC–MS/MS: high-performance liquid chromatography–mass spectrometry
HPMC: hydroxypropyl methylcellulose
LABA: long-acting agonists
LH: lung homogenates
LMWH: low-molecular-weight heparin
LPP: large porous particle
LT: leukotrienes
MMAD: mass median aerodynamic diameter
MCC: mucociliary clearance
MTK: montelukast
MNLC: montelukast-loaded nanostructured lipid carrier
NGI: next generation impactor
NLC: nanostructured lipid carriers
P_ao_: peak pressure of airway opening
P_app_: coefficient of apparent permeability
PCL: poly-ε-caprolactone
PEG: polyethylene glycol
PEI: polyethylenimine
PLA: polylactic acid
PLGA: poly­(lactic-co-glycolic) acid
pMDI: pressurized metered-dose inhalers
PVA: poly­(vinyl alcohol)
PVP: polyvinylpyrrolidone
SABA: short-acting agonists
SD: spray drying
SLN: solid lipid nanoparticles
TPP: tripolyphosphate
TNF: tumor necrosis factor
